# Replicating bacterium-vectored vaccine expressing SARS-CoV-2 Membrane and Nucleocapsid proteins protects against severe COVID-19-like disease in hamsters

**DOI:** 10.1038/s41541-021-00321-8

**Published:** 2021-03-30

**Authors:** Qingmei Jia, Helle Bielefeldt-Ohmann, Rachel M. Maison, Saša Masleša-Galić, Sarah K. Cooper, Richard A. Bowen, Marcus A. Horwitz

**Affiliations:** 1 Division of Infectious Diseases, Department of Medicine, 37-121 Center for Health Sciences, School of Medicine, University of California – Los Angeles, Los Angeles, CA USA; 2 School of Veterinary Science, University of Queensland, Brisbane, QLD Australia; 3 Department of Biomedical Sciences, Colorado State University, Fort Collins, CO USA; 4 Department of Microbiology, Immunology, and Pathology, Colorado State University, Fort Collins, CO USA

**Keywords:** Live attenuated vaccines, Live attenuated vaccines

## Abstract

To generate an inexpensive readily manufactured COVID-19 vaccine, we employed the LVS Δ*capB* vector platform, previously used to generate potent candidate vaccines against Select Agent diseases tularemia, anthrax, plague, and melioidosis. Vaccines expressing SARS-CoV-2 structural proteins are constructed using the LVS Δ*capB* vector, a highly attenuated replicating intracellular bacterium, and evaluated for efficacy in golden Syrian hamsters, which develop severe COVID-19-like disease. Hamsters immunized intradermally or intranasally with a vaccine co-expressing the Membrane and Nucleocapsid proteins and challenged 5 weeks later with a high dose of SARS-CoV-2 are protected against severe weight loss and lung pathology and show reduced viral loads in the oropharynx and lungs. Protection correlates with anti-Nucleocapsid antibody. This potent vaccine should be safe; inexpensive; easily manufactured, stored, and distributed; and given the high homology between Membrane and Nucleocapsid proteins of SARS-CoV and SARS-CoV-2, potentially serve as a universal vaccine against the SARS subset of pandemic causing β-coronaviruses.

## Introduction

The ongoing pandemic of COVID-19, caused by severe acute respiratory syndrome coronavirus 2 (SARS-CoV-2), has caused over 50 million cases and 1.2 million deaths as of this writing^1^. A safe and potent vaccine that protects against severe COVID-19 disease is urgently needed to contain the pandemic. Ideally, such a vaccine would be safe, inexpensive, rapidly manufactured, and easily stored and distributed, so as to be available quickly to the entire world population.

Previously, our laboratory developed a versatile plug-and-play single vector platform vaccine against Tier I Select Agents and emerging pathogens wherein a single live multi-deletional attenuated *Francisella tularensis* subsp. *holarctica* vector, LVS Δ*capB*, is used to express recombinant immunoprotective antigens of target pathogens^2,3^. The LVS Δ*capB* vector was derived via mutagenesis from live vaccine strain (LVS), a vaccine against tularemia originally developed in the Soviet Union via serial passage and subsequently further developed and tested in humans in the USA^4,5^. As with wild-type *F. tularensis*, LVS is ingested by host macrophages via looping phagocytosis, enters a phagosome, escapes the phagosome via a Type VI Secretion System, and multiplies in the cytoplasm^6–8^. While much more attenuated than LVS, the LVS Δ*capB* vector retains the capacity to invade and multiply in macrophages^9^. Using this platform technology, we have developed exceptionally safe and potent candidate vaccines that protect against lethal respiratory challenge with virulent strains of *Francisella tularensis*, *Bacillus anthracis*, *Yersinia pestis*, and *Burkholderia pseudomallei*, the causative agents of tularemia, anthrax, plague, and melioidosis, respectively^2,3^. These vaccines induce balanced humoral (antibody/neutralizing antibody in the case of anthrax toxin) and cell-mediated immune responses (polyfunctional CD4+ and CD8+ T-cells) against key immunoprotective antigens of target pathogens^3^. We have now used this platform to develop a COVID-19 vaccine.

SARS-CoV-2 has four structural proteins—the Spike (S) glycoprotein, Membrane (M), Envelope (E), and Nucleocapsid (N) proteins. Virtually all COVID-19 vaccines in development have focused on the S protein, which mediates virus entry into host cells via the angiotensin-converting enzyme 2 (ACE2) receptor^10,11^. These vaccines have been tested for efficacy most prominently in the rhesus macaque model of COVID-19. However, this is primarily a model of asymptomatic infection or mild disease, as animals typically do not develop either fever or weight loss; hence, vaccine efficacy in the rhesus macaque is quantitated primarily in terms of the vaccine’s impact on viral load rather than on clinical signs. In contrast, the golden Syrian hamster develops severe COVID-19-like disease, akin to that of hospitalized humans^12^, including substantial weight loss and quantifiable lung pathology.

Herein, we have employed the LVS Δ*capB* vector platform to construct six COVID-19 vaccines expressing one or more of all four structural proteins of SARS-CoV-2 and tested the vaccines for efficacy, administered intradermally (ID) or intranasally (IN), against a high dose SARS-CoV-2 respiratory challenge in hamsters. We show that the vaccine expressing the MN proteins, but not the vaccines expressing the S protein or its subunits in various configurations, is highly protective against severe COVID-19-like disease including weight loss and lung pathology, and that protection is highly correlated with serum anti-N antibody levels.

## Results

### Construction and verification of rLVS Δ*capB*/SCoV2 vaccine candidates

**Fig. 1:**
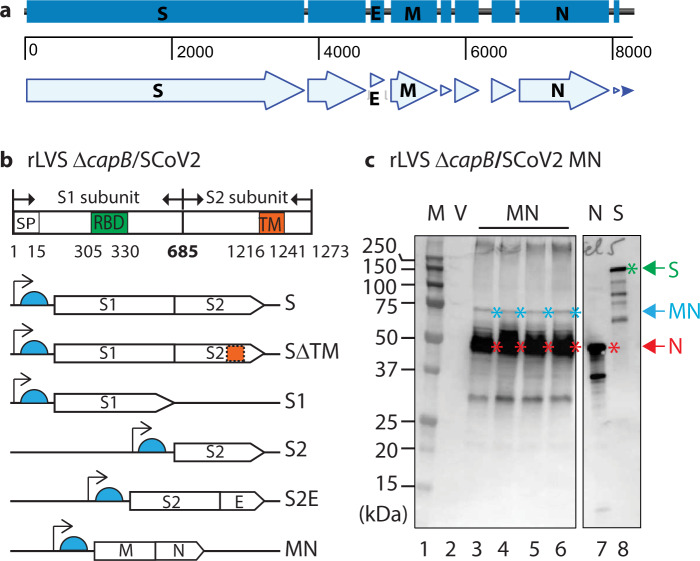
Construction of rLVS Δ*capB*/SARS-CoV-2 vaccines. **a** Schematic of SARS-CoV-2 genomic region encoding four major structural proteins, Spike (S) glycoprotein, Envelope (E), Membrane (M), and Nucelocapsid (N) protein. **b** Diagram of S protein and the antigen expression cassettes for S, SΔTM, S1, S2, fusion protein of S2 and E (S2E) and fusion protein of M and N (MN) downstream of the *F. tularensis bacterioferritin* (FTT1441) promoter (Pbfr) (thin black arrow) and Shine-Dalgarno sequence (light blue half circle). SP, signal peptide for S protein; RBD, receptor-binding domain; and TM, transmembrane domain. **c** Protein expression of rLVS Δ*capB*/SCoV2 MN. Total bacterial lysates of 4 clones of rLVS Δ*capB*/SCoV2 MN (lanes 3–6, as indicated at the bottom of the left panel) were analyzed by SDS-PAGE and Western blotting with an anti-SARS-CoV-1 guinea pig polyclonal antibody (BEI Resources, NR-10361), which readily detected the full-length MN (~75 kDa, less abundant), indicated by blue asterisks to the right of the protein bands, and the highly abundant breakdown product N protein (~46 kDa), indicated by red asterisks to the right of the protein bands. The anti-SARS-CoV-1 guinea pig polyclonal antibody also detected the N (red arrow and asterisk) and S (green arrow and asterisk) proteins of SARS-CoV-1 (lanes 7 and 8), which served as positive controls. V, LVS Δ*capB* vector (lane 2). The left and right panels are from the same gel (Supplementary Fig. 6). The sizes of the molecular weight markers (M) are labeled to the left of the panels.

We constructed six recombinant LVS Δ*capB* vaccines (rLVS Δ*capB*/SCoV2) expressing single, subunit or fusion proteins of four SARS-CoV-2 structural proteins: S^13^, E, M, and N (Fig. 1a). The S protein is synthesized as a single-chain inactive precursor of 1273 residues with a signal peptide (residues 1–15) and processed by a furin-like host proteinase into the S1 subunit that binds to host receptor ACE2^10^ and the S2 subunit that mediates the fusion of the viral and host cell membranes. S1 contains the host receptor-binding domain (RBD) and S2 contains a transmembrane domain (TM) (Fig. 1b, top panel). We constructed rLVS Δ*capB*/SCoV2 expressing S (with two Proline substitutions K986P/V987P) and, so as to express lower molecular weight constructs, SΔTM, S1, S2, and the fusion protein of S2 and E (S2E), and additionally, a vaccine expressing the fusion protein of M and N (MN) (Fig. 1b, bottom panels). A 3FLAG tag was placed at the N-terminus of the S, SΔTM, S1, and MN proteins. The antigen expression cassette of the SARS-CoV-2 proteins was placed downstream of a strong *F. tularensis* promoter (Pbfr) and a Shine-Dalgarno sequence (Fig. 1b) that we have used successfully to generate potent vaccines against *F. tularensis*, *Bacillus anthracis*, *Yersinia pestis*, and *Burkholderia pseudomallei*.

All six rLVS Δ*capB*/SCoV2 vaccine candidates, abbreviated as S, SΔTM, S1, S2, S2E, and MN, expressed the recombinant proteins from bacterial lysates. As shown in Fig. 1c, three protein bands—a minor 75 kDa, a major 46 kDa, and a minor 30 kDa band—were detected from lysates of 4 individual clones of the MN vaccine (Fig. 1c, lanes 3–6), but not from the lysate of the vaccine vector (lane 2) by Western blotting using guinea pig polyclonal antibody to SARS-CoV, which also detected the N and S protein of SARS-CoV (lanes 7 and 8, respectively). The 75-, 46-, and 30-kDa protein bands represent the full-length MN, the N, and degradation of the MN protein. The S, SΔTM, S1, S2, and S2E proteins were also expressed by the rLVS Δ*capB*/SCoV2 vaccines, as evidenced by Western blotting analysis using monoclonal antibody to FLAG to detect S, SΔTM, S1, and S2E (each with an N-terminus FLAG tag) and polyclonal antibody to SARS-CoV to detect non-tagged S2 protein (Supplementary Fig. 1a–d). Of note, SΔTM and S1 (Supplementary Fig. 1b) were expressed more abundantly than the full-length S protein (Supplementary Fig. 1a), possibly as a result of the removal of the TM domain and reduced size of the protein.

### Study of vaccine efficacy against SARS-CoV-2 challenge in the hamster model

**Fig. 2:**
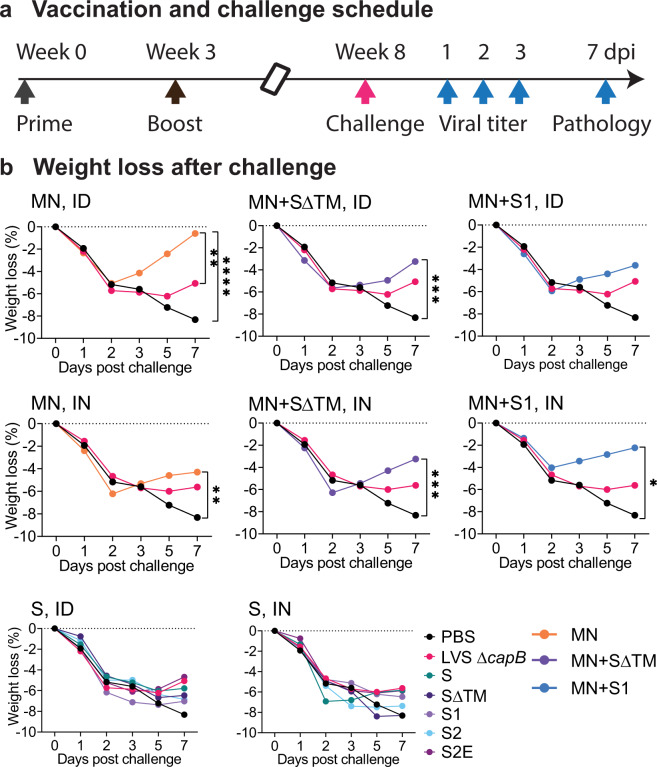
Experimental schedule and weight loss after challenge. **a** Experiment schedule. Golden Syrian hamsters (8/group, equal sex) were immunized ID or IN twice (Week 0 and 3) with rLVS Δ*capB/*SCoV2 vaccines, singly and in combination (MN + SΔTM; MN + S1); challenged IN 5 weeks later (Week 8) with 10^5^ pfu of SARS-CoV-2 (2019-nCoV/USA-WA1/2020 strain), and monitored closely for clinical signs of infection including weight loss. Single vaccines expressed the S, SΔTM, S1, S2, S2E, or MN proteins, as indicated. Control animals were sham-immunized (PBS) or immunized with the vector (LVS Δ*capB*) only. All hamsters were assayed for oropharyngeal viral load at 1, 2, and 3 days post challenge (dpi). Half of the hamsters (*n* = 4/group) were euthanized at 3 days post challenge for lung viral load analysis and half (*n* = 4/group) were monitored for weight loss for 7 days and euthanized at 7 days post challenge for lung histopathology evaluation. **b** Weight loss after challenge. Data are mean % weight loss from 0 days post challenge. *P < 0.05; **P ≤ 0.01; ***P < 0.001; ****P ≤ 0.0001 comparing means on Day 7 post challenge by repeated measure (mixed) analysis of variance model. Sham vs. MN: *P* < 0.0001, ID route; *P* < 0.01, IN route. The standard errors were omitted in the graphs for clarity.

We immunized Syrian hamsters ID or IN twice, 3 weeks apart, with the six rLVS Δ*capB*/SCoV2 vaccines—S, SΔTM, S1, S2, S2E, and MN—singly and in combination (MN + SΔTM; MN + S1). Five weeks later, we challenged the animals with a high dose of SARS-CoV-2 administered IN, and then closely monitored them for clinical signs of infection including weight loss. Animals immunized with PBS (Sham) or with the vector LVS Δ*capB* served as controls. At 1, 2, and 3 days post challenge, oropharyngeal swabs were collected daily and assayed for viral load. At 3 and 7 days post challenge, half of the animals in each group were euthanized and evaluated for lung viral load and lung histopathological changes, respectively (Fig. 2a).

### MN vaccine protects against SARS-CoV-2-induced weight loss in the hamster model

As shown in Fig. 2b (top and middle panels), hamsters immunized either ID (top panels) or IN (middle panels) with the MN vaccine, alone or in combination with the SΔTM or S1 vaccines, were significantly protected against severe weight loss after high dose SARS-CoV-2 IN challenge [*P* < 0.0001, *P* < 0.01, and *P* < 0.0001 for Sham vs MN administered ID, IN, or ID/IN (either ID or IN), respectively (Day 7) and *P* < 0.0001 for Sham vs. all MN vaccine groups administered ID/IN] (Supplementary Table 1a). All animals lost ~5% of their total body weight during the first 2 days after challenge; however, hamsters immunized with the MN vaccine, alone or in combination with the SΔTM or S1 vaccine, began to recover from the weight loss starting on Day 3, such that hamsters immunized with MN, MN + SΔTM, and MN + S1 vaccines ID regained 81%, 43%, and 38% (mean ± SE 54% ± 11%) of the lost weight by Day 7 and hamsters immunized with MN, MN + SΔTM, and MN + S1 vaccines IN, regained 63%, 47%, and 70% (mean ± SE 60% ± 5.6%) of the lost weight by Day 7, whereas sham-immunized animals continued to lose weight until euthanized on Day 7, by which time they had lost a mean of 8% of their total body weight. Hamsters immunized with the vector control continued to lose weight until Day 5 and then exhibited a small partial recovery, possibly reflecting a small beneficial non-specific immunologic effect as has been hypothesized for BCG and other vaccines^14–17^. In contrast to hamsters immunized with the MN vaccine, hamsters immunized with the S, SΔTM, S1, S2, or S2E vaccines, administered ID or IN, were not protected against severe weight loss (Fig. 2b, bottom panels).

### MN vaccine protects against severe lung pathology in the hamster model

**Fig. 3:**
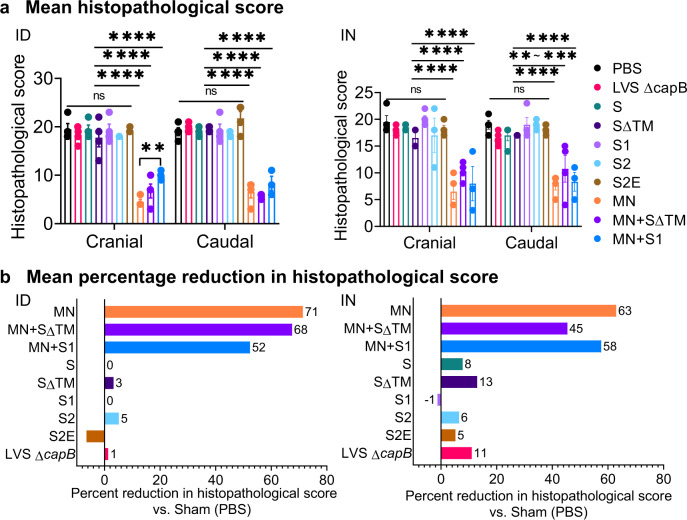
Lung histopathology on Day 7 after SARS-CoV-2 IN challenge. Hamsters (*n* = 4, equal sex) were immunized ID or IN as described in Fig. 2 and euthanized at 7 days post challenge for histopathologic examination of their lungs. **a** Cranial and caudal lung histopathology post challenge in hamsters immunized ID (left) or IN (right) were separately scored on a 0–5 or 0–4 scale for overall lesion extent, bronchitis, alveolitis, pneumocyte hyperplasia, vasculitis, and interstitial inflammation; the sum of the scores for each lung are shown (mean ± SE). The histopathological score evaluation was performed by a single pathologist blinded to the identity of the groups. Each symbol represents one animal. Data are mean ± SE. **P < 0.01; ***P < 0.001; ****P < 0.0001 by two-way ANOVA with Tukey’s multiple comparisons (GraphPad Prism 8.4.3); ns, not significant. **b** The mean percentage reduction in the combined cranial and caudal lung histopathology score compared with Sham (PBS)-immunized animals was calculated for each vaccine.

**Fig. 4:**
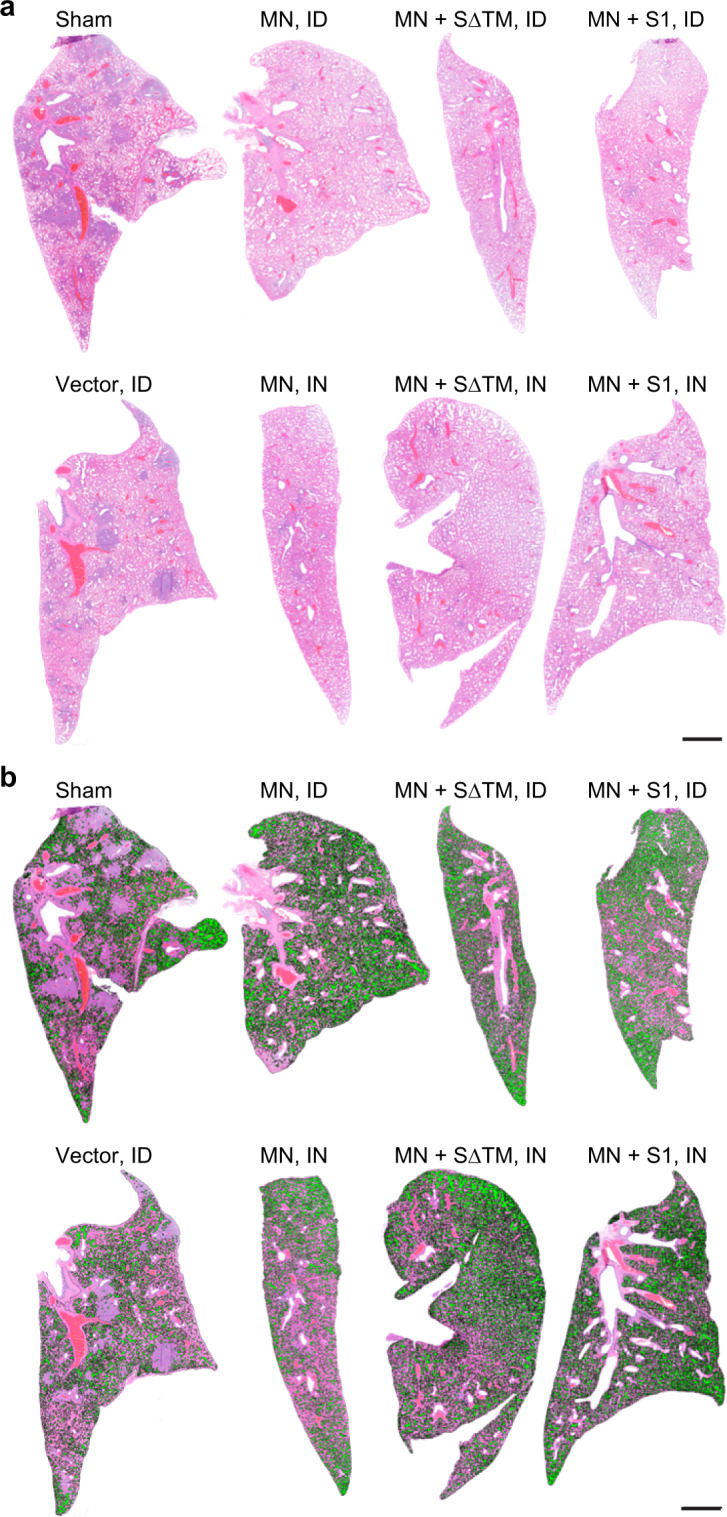
Impact of MN vaccines on histopathology and percent alveolar air space. The selected images were obtained from a lung of a hamster scoring near the mean histopathological score of its immunization group, as indicated. **a** Histopathology (H&E stained lung sections). **b** Percent alveolar air space. The percent alveolar air space for each lung shown is as follows: Sham, 23.6%; MN vaccine administered intradermally [MN (ID)], 33.4%; MN + SΔTM (ID), 31.6%; MN + S1 (ID), 31.2%; LVS Δ*capB* vector [Vector (ID)], 20.8%; MN vaccine administered intranasally [MN (IN)], 25.5%; MN + SΔTM (IN), 31.4%; and MN + S1 (IN), 35%. Scale bars = 2 mm.

To evaluate vaccine efficacy against SARS-CoV-2-induced lung disease, we assessed cranial and caudal lung histopathology on Day 7 post challenge, which peaks in unvaccinated animals at this time point (unpublished observation)^18^. As shown in Figs. 3a, 4a, and Supplementary Tables 2 and 3, hamsters immunized either ID or IN with the MN vaccine, alone (MN) or in combination with SΔTM or S1, were consistently protected against severe lung pathology after high dose SARS-CoV-2 IN challenge (*P* < 0.0001 vs. sham-immunized hamsters for all MN containing groups, whether administered ID or IN; *P* < 0.0001 vs. vector control for all MN groups when administered ID and *P* < 0.01–*P* < 0.0001 vs. vector control for all MN groups when administered IN) (Supplementary Table 1b). Compared with sham-immunized hamsters, the histopathology score in the cranial and caudal lungs of hamsters vaccinated with the MN vaccine was reduced on average by 71% when administered ID and 63% when administered IN. In contrast, hamsters immunized with one of the five S protein vaccines were not significantly protected against severe lung pathology whether the vaccines were administered ID or IN (Fig. 3b).

**Fig. 5:**
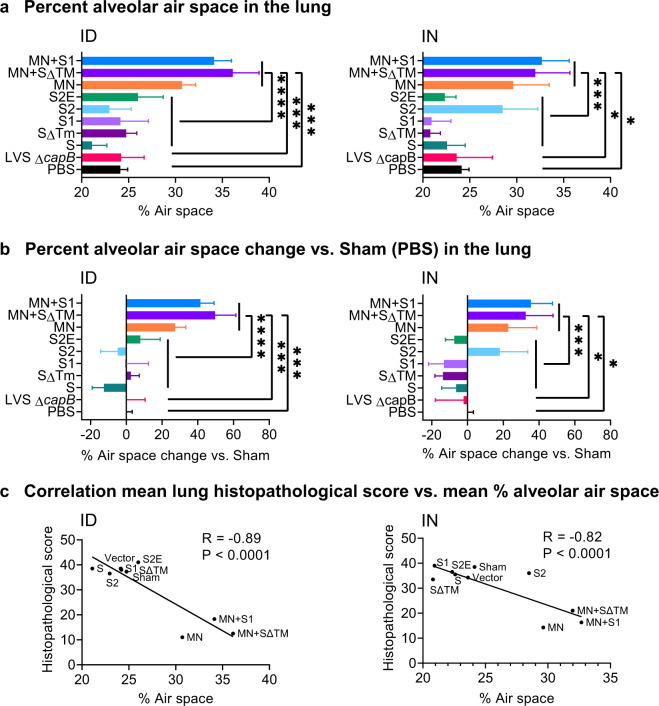
Percent alveolar air space and correlation between lung histopathological score and percent alveolar air space. **a** Percent alveolar air space was quantitated as described in “Methods” at Day 7 post 10^5^ CFU SARS-CoV-2 intranasal challenge in lungs of hamsters immunized ID (left) or IN (right) with the indicated vaccines. **b** Percent alveolar air space vs. Sham (PBS) [(Air space of immunized animal − Mean air space of Sham)/Mean air space of Sham]. **a**, **b** Shown are means + SE. Animals immunized with PBS (Sham), LVS Δ*capB* (Vector), the S vaccines (S, SΔTM, S1, S2, and S2E), and the MN vaccines (MN, MN + SΔTM, and MN + S1) were compared by ANOVA (JMP 15.0); *P < 0.05; ***P < 0.001; and ****P < 0.0001. **c** Correlation between mean lung (cranial and caudal) histopathological score and mean percent alveolar air space for ID (left) and IN (right) vaccination route (Prism 9.0.0). In addition, the correlation between lung histopathological score and mean percent alveolar air space for individual animals is *R* = −0.74 (*P* < 0.0001) (ID) and *R* = −0.80 (*P* < 0.0002) (IN) (JMP 15.0).

In addition to a conventional histopathological assessment, as an independent measure of lung inflammation, we quantitated the percent of lung tissue comprising alveolar air space. Consistent with the histopathological assessment, hamsters immunized ID or IN with the MN vaccines (MN, MN + SΔTM, MN + S1) had significantly greater percent alveolar air space than hamsters immunized with PBS, the LVS Δ*capB* vector control, or non-MN vaccines (Figs. 4b and 5). The percent alveolar air space correlated negatively with lung histopathological score (*R* = −0.89, *P* < 0.0001 ID; *R* = −0.82, *P* < 0.0001 IN) (Fig. 5c).

### MN vaccine protects against SARS-CoV-2 viral replication in the oropharynx and lungs of hamsters

**Fig. 6:**
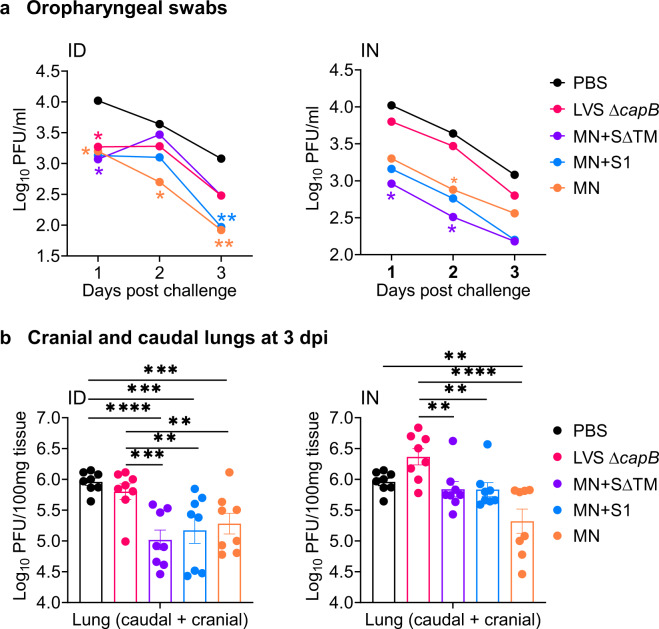
Viral load in oropharyngeal swabs and cranial and caudal lungs 3 days post challenge. Hamsters were immunized ID or IN as described in Fig. 2. **a** Oropharyngeal swabs were collected at 1, 2, and 3 days post challenge and assayed for viral load by plaque assay. Data are mean Log_10_ PFU per ml. The standard errors were omitted in the graphs for clarity. *P < 0.05; **P < 0.01 (color coded to each vaccine) vs. Sham (PBS) by repeated measure analysis of variance. **b** Cranial and caudal lung homogenates were prepared at 3 days post challenge and assayed for viral titer. Data are mean ± SE of PFU per 100 mg of homogenized tissue, as indicated. Each symbol represents one animal. **P < 0.01; ***P < 0.001; ****P < 0.0001 by two-way ANOVA with Dunnett multiple comparisons (GraphPad Prism 8.4.3).

To examine the impact of vaccines on viral replication, we collected oropharyngeal swabs of all hamsters (*n* = 8/group) on Days 1, 2, and 3 post challenge and assayed viral load by plaque assay. Hamsters immunized ID or IN with MN alone, or in combination with SΔTM or S1, showed significantly reduced viral titers in the oropharynx. Specifically, compared with sham-immunized animals, hamsters immunized ID with MN showed a 0.8 ± 0.4, 1.0 ± 0.4, and 1.2 + 0.4 log_10_ PFU (Plaque Forming Units) reduction (mean ± SE) in viral load at Days 1, 2, and 3 post challenge, respectively (*P* = 0.04, 0.02, and 0.004, resp.); hamsters immunized ID with MN + SΔTM or MN + S1 also showed significant reductions in viral titer compared with sham-immunized animals on Day 1 (*P* < 0.05 for both vaccines) and, for MN + S1, on Day 3 (*P* < 0.01) post challenge (Fig. 6a, left graph). Animals immunized IN with MN vaccines (MN, MN + SΔTM, MN + S1) also showed reduced viral load compared with sham- and vector-immunized animals on Days 1–3 post challenge, on average 0.8 ± 0.3, 0.8 ± 0.3, and 0.6 ± 0.3 log_10_ PFU fewer than Sham on Days 1–3 post challenge (*P* < 0.02 for all MN vaccines vs. Sham on Days 1 and 2 post challenge) (Fig. 6a, right graph). All MN vaccines combined, whether administered ID or IN, showed mean reductions compared with Sham of 0.9 ± 0.3, 0.6 ± 0.3, and 0.8 ± 0.3 log_10_ PFU on Days 1, 2, and 3, respectively (*P* < 0.01, *P* < 0.05, and *P* < 0.01, resp.). In contrast, hamsters immunized with the S protein vaccines (S, S∆TM, S1, S2, and S2E) did not show significantly reduced viral titers compared with sham-immunized animals whether the vaccines were administered ID or IN (Supplementary Fig. 2).

To evaluate viral replication in the lungs, we assayed cranial and caudal lungs for viral load on Day 3 post challenge, which peaks at this time point in unvaccinated animals (unpublished observation)^18^. Hamsters immunized ID with the MN vaccine, alone or in combination with the SΔTM or S1, showed significantly reduced viral loads in their cranial and caudal lungs compared with sham- or vector-immunized animals (Fig. 6b, left panel). Hamsters immunized ID with the MN vaccines as a group showed a mean reduction of 0.8 ± 0.1 log_10_ PFU compared with Sham (*P* < 0.0001). In contrast, hamsters immunized ID with the S (S, SΔTM, S1, S2, S2E) protein vaccines did not show reduced viral loads in their cranial and caudal lungs (data not shown). Similar results were observed in hamsters immunized IN (Fig. 6b, right panel).

### MN expressing vaccines induce antibody to N protein with a TH1 bias

**Fig. 7:**
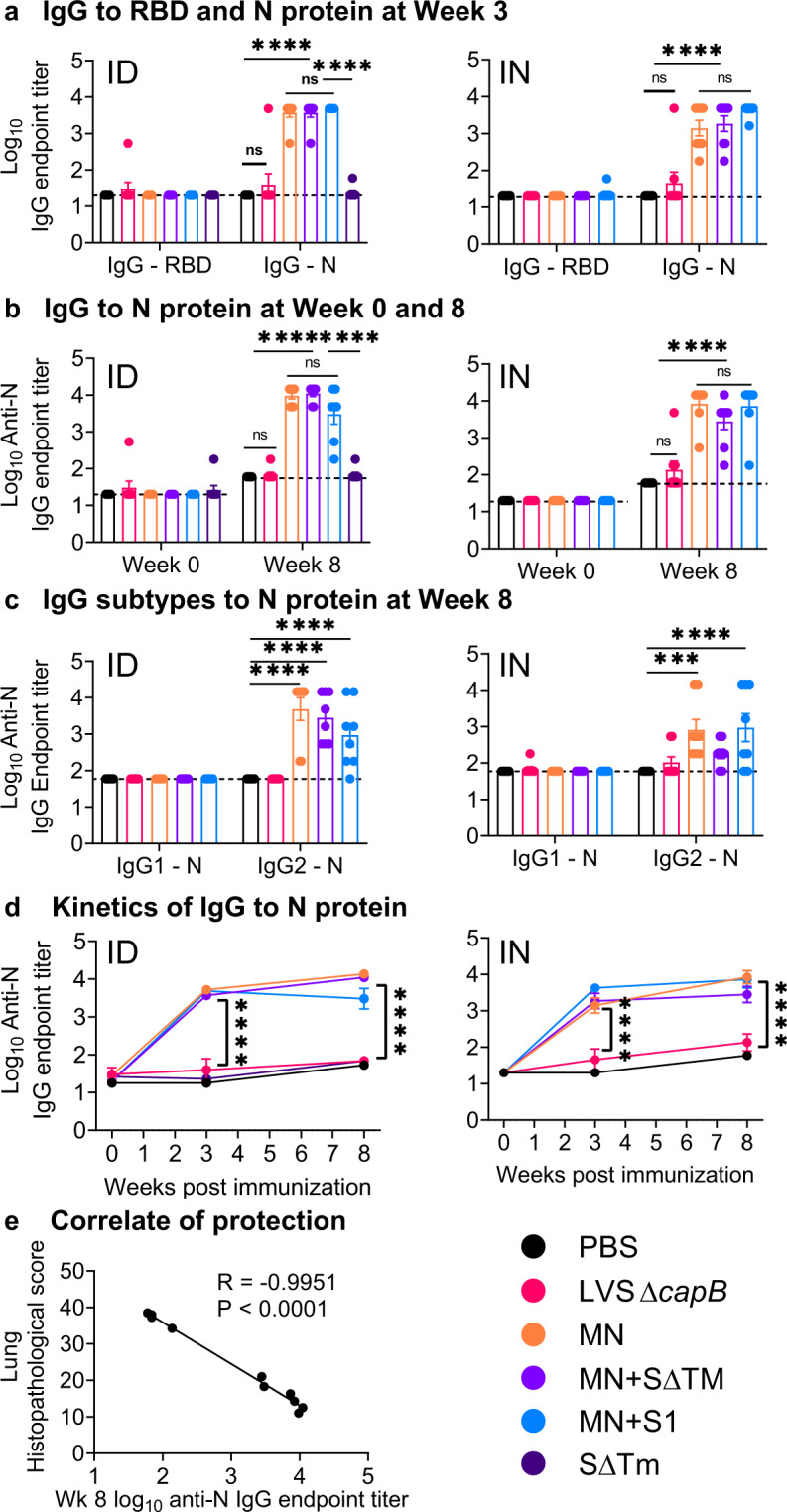
Humoral immune response and correlate of protection. Hamsters were immunized ID or IN as described in Fig. 2. **a** Sera were evaluated for IgG specific to S RBD and N protein three weeks after a single immunization ID (left) or IN (right). **b** Sera were evaluated for IgG specific to N protein just prior to immunization at Week 0 and just prior to challenge at Week 8 in hamsters immunized ID (left) or IN (right). **c** Sera were evaluated for IgG subtypes (IgG1 and IgG2) specific to N protein at Week 8 in hamsters immunized ID (left) or IN (right). **a**–**c**, each symbol represents one animal. **d** The antibody titers displayed in (**a**) and (**b**) are plotted over time. **a**–**d** Data are mean ± SE. ***P < 0.001; ****P < 0.0001; ns, not significant by two-way ANOVA with Tukey’s multiple comparisons (GraphPad Prism 8.4.3). **e** Correlation between mean IgG N titer at Week 8 and mean lung histopathology score on Day 7 post challenge (sum of cranial and caudal lung as shown in Fig. 3a) for all groups (ID and IN).

To assess antibody responses to SARS-CoV-2 proteins expressed by the vaccine, we analyzed antibodies to the RBD of the S protein and to the N protein (Fig. 7). As expected, sera from sham-immunized hamsters lacked antibody to either antigen as did sera from all but a small minority of LVS Δ*capB* vector-immunized hamsters (Fig. 7a–c). In contrast, sera from hamsters immunized once with the MN vaccine, alone or in combination with the SΔTM or S1 vaccine, showed high levels of N specific IgG, whether immunized ID or IN, at 3 weeks post-immunization (Fig. 7a), which somewhat increased at Week 8, 5 weeks after the second immunization at Week 3 (Fig. 7b), displaying a TH1 type bias, with IgG2 dominating the response (Fig. 7c). Differences in serum anti-N IgG titers between hamsters immunized with the MN vaccine, alone or in combination with S protein vaccines, and sham- or vector-immunized hamsters were highly significant at both Week 3 and Week 8 (*P* < 0.0001) (Fig. 7d). Surprisingly, hamsters immunized with S protein vaccines did not show anti-RBD antibody at Week 3 (Fig. 7a); however, at Week 8, 5 weeks after the second immunization, RBD-specific IgG was detected in animals immunized with vaccines expressing the S antigen, especially in animals immunized ID with the MN + SΔTM vaccine (Supplementary Fig. 3). Nevertheless, the RBD-specific IgG antibody did not confer SARS-CoV-2 neutralizing activity (data not shown). In mice immunized at Weeks 0 and 3 with second-generation vaccines expressing MN in combination with S1 or SΔTM, serum obtained at Week 4 showed anti-RBD antibody as well as anti-N antibody (Supplementary Fig. 4). Anti-N IgG antibody displayed a TH1 type bias both in hamsters (Fig. 7c), where IgG2 dominated the IgG response, and in mice, where IgG2a dominated the IgG response (Supplementary Fig. 4). This TH1 bias was also reflected by murine splenocyte secretion of IFN-γ in response to S and N peptides (Supplementary Fig. 5).

### Serum anti-N antibody correlates with protection in hamsters

We assessed the correlation coefficient between serum anti-N IgG antibody just before challenge at Week 8 and lung (cranial + caudal) histopathological scores at Day 7 post challenge by linear regression analysis. Anti-N antibody was highly and inversely correlated with histopathology score (*R* = −0.9951, *P* < 0.0001) (Fig. 7e). This antibody, which does not neutralize SARS-CoV-2 (data not shown), likely is not itself protective but instead correlates with a protective T-cell response such as that shown in Supplementary Fig. 5.

## Discussion

We show that a replicating LVS Δ*capB*-vectored COVID-19 vaccine, rLVS Δ*capB*/SCoV2 MN, that expresses the SARS-CoV-2 M and N proteins, protects against COVID-19-like disease in the demanding golden Syrian hamster model. The vaccine significantly protects against weight loss and severe lung pathology, the two major clinical endpoints measured, and significantly reduces viral titers in the oropharynx and lungs. The vaccine was protective after either ID or IN administration.

Surprisingly, of the six vaccines expressing one or more of the four SARS-CoV-2 structural proteins, only the vaccine expressing the MN proteins was protective. Such a vaccine has the potential to provide cross-protective immunity against the SARS subgroup of β-coronaviruses including potential future pandemic strains. While the S protein shows only 76% sequence identity between SARS-CoV and SARS-CoV-2, the M and N proteins each show 90% identity^19^. In an analysis of T-cell epitopes in humans recovered from COVID-19, the M and N antigens together accounted for 33% of the total CD4+ T-cell response (21 and 11% for M and N, respectively) and 34% of the total CD8+ T-cell response (12 and 22% for M and N, respectively), an amount exceeding the 27 and 26% CD4 and CD8 T-cell responses, respectively, of the S protein^20^. Considering that the M and N proteins are more conserved than the S protein among the SARS subgroup of β-coronaviruses and the dominant influence of the M and N proteins in the T-cell immune response, the MN vaccine has potential for universal protection against this group of especially severe pandemic strains.

We evaluated our vaccines in the hamster model of SARS-CoV-2 infection because of its high similarity to serious human COVID-19 disease, which likely reflects at least in part the high genetic similarity of the hamster and human ACE2 receptor–S protein interface. A modelling of binding affinities showed that the hamster ACE2 has the highest binding affinity to SARS-CoV-2 S of all species studied with the exception of the human and rhesus macaque^12^.

In our previous studies of vaccines utilizing the LVS Δ*capB* vector platform, three immunization doses consistently yielded superior efficacy to two doses. Here, given the urgency for a COVID-19 vaccine and the desire to simplify the logistics of vaccine administration, we opted to test only two immunizations, while still maintaining a reasonably long immunization-challenge interval (5 weeks after the second immunization). Future studies will examine if three doses are superior to two and the longevity of immunoprotection.

Generally speaking, vaccine efficacy in a relevant animal model of disease is the best predictor of vaccine efficacy in humans. That non-human primates (NHPs) challenged with SARS-CoV-2 develop only mild disease or remain asymptomatic brings into question the utility of this animal model as a predictor of COVID-19 vaccine efficacy in humans. Nevertheless, most published studies on vaccine efficacy have been conducted in NHPs^21–26^; the absence of quantifiable clinical symptoms limited these studies to measuring differences in viral load between immunized and control animals. In contrast to NHPs, SARS-CoV-2 challenged hamsters develop quantifiable clinical symptoms, especially weight loss and lung pathology, akin to humans seriously ill with COVID-19^12,18,27^. Since the major utility of a vaccine is in preventing serious infection and death, the hamster is a highly relevant animal model for assessing COVID-19 vaccine efficacy, and studies in hamsters can be conducted at a fraction of the cost, complexity, human and facility resources, and ethical concerns of studies in NHPs.

Many types of vaccines are being developed against COVID-19 including DNA, RNA, and protein/adjuvant vaccines, non-replicating and replicating viral-vectored vaccines, whole inactivated virus vaccines, and virus-like particles. To our knowledge, ours is the only vaccine comprising a replicating bacterial vector. Replicating vaccines are among the most successful vaccines in history with a reputation for inducing comprehensive immune responses and long-lasting immunity^28^.

Our LVS Δ*capB*-vectored vaccine platform offers several advantages including (1) low toxicity; (2) ability to express multiple antigens of target pathogens from two different loci; (3) balanced immunogenicity—B-cell and T-cell (TH1 type); (4) ease of administration by multiple routes (intradermal, subcutaneous, intramuscular, intranasal, oral, etc.); (5) no animal products in contrast to viral-vectored vaccines grown in cell culture; (6) no need for adjuvant; (7) no pre-existing immunity as with adenoviruses; (8) low cost of manufacture as extensive purification is not required, in contrast to RNA, protein/adjuvant and viral-vectored vaccines; (9) ease of large scale manufacture via bacterial fermentation in simple broth culture; and (10) after lyophilization, convenient storage and distribution at refrigerator temperatures. These last three advantages are particularly important with respect to making a COVID-19 vaccine available rapidly and cheaply to the entire world’s population.

Also of particular importance with respect to vaccine distribution and acceptance is the potential of our vaccine for oral administration. Oral administration of the LVS vaccine to mice and monkeys has demonstrated both immunogenicity and efficacy against lethal *F. tularensis* respiratory challenge, and oral immunization of human volunteers with LVS has been reported to induce antigen-specific antibody responses at least as rapidly as intradermal immunization^29–32^.

Safety is always a major consideration in vaccine development, especially so in the case of replicating vaccines. In our vaccine’s favor, its much less attenuated parent (LVS) was already considered safe enough to justify extensive testing in humans, including recently, and it has demonstrated safety and immunogenicity^5,33–39^. LVS has two major attenuating deletions and several minor ones^40^. As many as 60 million Russians were reportedly vaccinated against tularemia with the original LVS strain^41^, and over 5000 laboratory workers in the United States have been vaccinated with the modern version of LVS by scarification^5^. Our further attenuation of LVS by introduction of the *capB* mutation reduced its virulence in mice by the IN route by >10,000-fold^9^. Hence, rLVS Δ*capB*/SCoV2 MN and other LVS Δ*capB*-vectored vaccines are anticipated to be exceedingly safe.

Correlates of protective immunity to COVID-19 are not well understood. Almost all of the vaccines in development are centered on generating immunity to the S protein—especially neutralizing antibody to this protein. However, neutralizing antibody alone may not be sufficient for full protection; vaccines generating strong neutralizing antibody responses against SARS-Co-V were not necessarily highly protective, especially in ferrets, which exhibit SARS disease more akin to that in humans^42,43^. T-cell responses may be as or more important. T-cell responses were demonstrated to be required to protect against clinical disease in SARS-CoV challenged mice and adoptive transfer of SARS-CoV specific CD4 or CD8 T-cells into immunodeficient mice infected with SARS-CoV lead to rapid viral clearance and disease amelioration^44^.

Our S protein vaccines were ineffective, reflecting poor immunogenicity, as evidenced by the variable but generally low RBD-specific IgG in the blood of hamsters (Supplementary Fig. 3); the rapid decline of antibody titer in mice (data not shown); and the negligible neutralization antibody titers (<10) in the blood of all hamsters just before challenge (data not shown) including the few with especially high IgG anti-RBD titers. Possible explanations for the poor immunogenicity include instability of the S protein expressed by the vector, differences in antigen presentation between protein secreted by the vector and protein displayed on the virus surface, and differences in post-translational modification (i.e., glycosylation) between protein expressed via virus infection of eukaryotic cells and protein expressed by an intracellular prokaryotic vector. Possibly, alternative expression of the S protein, for example display on the bacterial surface, as reported for the S protein of SARS-CoV^45^, or expression via a eukaryotic promoter would improve immunogenicity. If so, this would allow immune responses to the S protein to contribute to the already substantial protective efficacy provided by immune responses to the M and N proteins.

Our replicating bacterial vaccine expressing the M and N proteins has demonstrated safety and efficacy in an animal model of severe COVID-19-like disease. If its safety and efficacy are reproduced in humans, the vaccine has potential to protect people from serious illness and death. Considering the ease with which our vaccine can be manufactured, stored, and distributed, it has the potential to play a major role in curbing the COVID-19 pandemic.

## Methods

### Ethics statement

Hamsters and mice were used according to protocols approved by the Institutional Animal Care and Use Committees of Colorado State University (CSU) and UCLA, respectively.

### Cells, virus, and bacteria

Vero E6 cells (ATCC #CCL-81, Manassas, VA, USA) were grown in Dulbecco’s modified Eagle medium (DMEM) with high glucose (Millipore Sigma, St. Louis, MO, USA). SARS-CoV-2 virus (2019-nCoV/USA-WA1/2020 strain) was acquired through the NIH NIAID Biodefense and Emerging Infections Research Resources Repository (BEI Resources, NR-52281, Lot 700033175), passaged three times in Vero E6 cells, and stocks frozen in DMEM supplemented with 10% fetal bovine serum. We sequenced the virus on receipt and three times thereafter pre-challenge. Compared with the originally received strain, the pre-challenge strain had 5 mutations in four proteins as follows: S protein: D215H, R685H; N protein: S194T; M protein T7I; nsp12 protein: M135R. The virus titer was determined by plaque assay as described previously^46^ and below. *F. tularensis* Live Vaccine Strain with a deletion in *capB* (LVS Δ*capB*) was constructed as described by us previously^9^. Live attenuated recombinant LVS Δ*capB* expressing SARS-CoV-2 antigens (rLVS Δ*capB*/SCoV2 and rLVS Δ*capB*::MN/SCoV2) were constructed as described below. Stocks of LVS Δ*capB* vector, rLVS Δ*capB*/SCoV2, and rLVS Δ*capB*::MN/SCoV2 vaccines were prepared in broth medium. Briefly, the bacteria were inoculated in Medium T broth^47^ at an initial optical density (OD) of 0.003–0.005 at 600 nm absorbance, grown overnight at 37 °C with shaking, harvested by centrifugation at 5000 × *g* for 20 min, washed twice with sterile normal saline, suspended in 20% glycerol—normal saline solution, and frozen in 0.25 ml aliquots at −80 °C until use. The titers of bacterial stocks were determined immediately before and periodically after freezing by spotting 0.05 ml of 10-fold serial diluted bacterial suspension onto Chocolate agar plates supplemented with or without kanamycin (7.5 µg/ml); and the plates were incubated at 37 °C for 3–5 days before colony forming units (CFU) were counted.

### Proteins, antibodies, and heat-inactivated viruses and bacteria

We obtained the following reagents through BEI Resources: SARS coronavirus S glycoprotein with deleted TM domain, SΔTM, recombinant from Baculovirus (NR-722); SARS-CoV-2 S glycoprotein (stabilized), recombinant from Baculovirus (NR-52396; NR-52308); SARS-CoV-2 S glycoprotein RBD with C-Terminal histidine tag, recombinant from HEK293T Cells (NR-52946); SARS coronavirus N glycoprotein, recombinant from *E. coli* (NR-699); SARS-CoV-2 N protein N-terminal RNA binding domain with N-terminal histidine tag, recombinant from *E. coli* (NR-53246); SARS-CoV-2 S glycoprotein peptide array (NR-52402); SARS-CoV-2 N protein peptide array (NR-52404); heat-inactivated SARS-CoV-2, isolate USA-WA1/2020 (NR-52286); and guinea pig polyclonal anti-SARS coronavirus antibody (NR-10361). Monoclonal anti-FLAG M2 HRP antibody was purchased from Millipore Sigma (St. Louis, MO). Stocks of heat-inactivated LVS Δ*capB* (HI-LVS) were prepared as described previously^9^.

### Generation of rLVS Δ*capB*/SCoV2 vaccines

Six live attenuated recombinant LVS Δ*capB* expressing SARS-CoV-2 structural proteins (rLVS Δ*capB*/SCoV2) S, SΔTM, S1 subunit, S2 subunit, or fusion proteins of S2E (S2 subunit fused to E protein) and MN (M protein fused to N) from a shuttle plasmid were constructed by electroporating a shuttle plasmid carrying a SARS-CoV-2 antigen expression cassette downstream of the *Francisella* bacterioferritin (bfr) promoter (Pbfr) into rLVS Δ*capB*, as we published previously^3^. Briefly, to construct a shuttle plasmid expressing the S protein (protein id QIH55221.1), we codon-optimized a gene (Genebank MT152824) encoding the full-length SARS-CoV-2 S protein with two stabilizing proline substitutions at the S2 fusion machinery (K986P and V987P)^48,49^ for expression in LVS and had it synthesized by Atum.com. Similarly, genes encoding fusion proteins of S2E and MN, linked by a flexible linker (GGSG), were codon-optimized and synthesized by Atum.com. Subsequently, we cloned the codon-optimized DNA for S, S2E, and MN with an N-terminal 3FLAG tag into the pFNL-derived shuttle plasmid downstream of the Pbfr promoter to generate pFNL/Pbfr-N3F-S, pFNL/Pbfr-N3F-S2E, and pFNL/Pbfr-N3F-MN. We further mutated the S protein expression cassette in the pFNL/Pbfr-N3F-S plasmid and generated pFNL/Pbfr-N3F-SΔTM, pFNL/Pbfr-N3F-S1, and pFNL/Pbfr-S2 by using the QuikChange Lightning Site-Directed Mutagenesis Kit (Agilent Technologies, https://www.agilent.com) and the following primer pairs: TGAGGTTAAGGATCCACTAGCTCGTTTCAAA and TTGTTCGTATTTTCCAAGTTCTTGTAGATCTATTAAA for generating pFNL/Pbfr-N3F-SΔTM; TGAGGTTAAGGATCCACTAGCTCGTTTCAAA and TCTTGCGCGACGAGGACTATTTGTCTGTGT for generating pFNL/Pbfr-N3F-S1; and TCAGTAGCATCACAATCGATTATAGCTTATACAA and CATTACGTACCTCCTATTGTTACCTCCATTATTTA for generating pFNL/Pbfr-S2. After verifying the nucleotide sequences for the SARS-CoV-2 protein expression cassette by restriction analysis and nucleotide sequencing, we electroporated the pFNL plasmid that carries a kanamycin-resistance gene into LVS Δ*capB* electro-competent cells; selected recombinant clones (rLVS Δ*capB*/SCoV2 S, SΔTM, S1, S2, S2E, and MN) on Chocolate agar plates supplemented with kanamycin (7.5 µg/ml); and verified kanamycin-resistant clones by nucleotide sequencing of the antigen expression cassette and by western blotting for protein expression.

### Generation of second-generation multi-antigenic rLVS Δ*capB*::MN/SCoV2 vaccines

We also generated second-generation rLVS Δ*capB*::MN/SCoV2 vaccine candidates expressing the MN fusion protein from an antigen expression cassette integrated at the deleted *capB* locus in the chromosome and expressing the SΔTM, S1, or S2 protein from an antigen expression cassette located in a shuttle plasmid DNA. To construct rLVS Δ*capB* expressing the MN antigen from the chromosome, we amplified three DNA fragments—one encoding the antigen expression cassette Pbfr-N3F-MN, one homologous to the upstream region of the *capB* gene in the LVS Δ*capB* chromosome, and one homologous to the downstream region of the *capB* gene—by PCR using pFNL/Pbfr-N3F-MN and LVS Δ*capB* genomic DNA as templates for the first, second, and third DNA fragments, respectively. We assembled the three DNA fragments by using the Gibson Assembly kit (NEB, Ipswich, MA), cloned the assembled DNA into the pMP590 integration plasmid^50^ that contains a kanamycin-resistance gene and a sucrose suicide gene, and verified the DNA sequence by restriction analysis and nucleotide sequencing. The resultant recombinant DNA, pMP/Pbfr-N3F-MN, was integrated into the *capB* locus by allelic exchange on Chocolate agar supplemented with kanamycin (7.5 µg/ml) followed by selection on Chocolate agar supplemented with 8% sucrose agar to generate marker-free rLVS Δ*capB*::MN. The chromosome integration of the MN expression cassette at the *capB* locus was verified by PCR for DNA integration and by Western blotting for MN fusion protein expression using monoclonal anti-FLAG M2 HRP antibody and/or guinea pig polyclonal antibody to SARS coronavirus. Subsequently, we introduced pFNL/Pbfr-N3F-SΔTM, pFNL/Pbfr-N3F-S1, and pFNL/Pbfr-S2 into rLVS Δ*capB*::MN to generate rLVS Δ*capB*::MN/SΔTM, rLVS Δ*capB*::MN/S1 and rLVS Δ*capB*::MN/S2 expressing MN plus SΔTM, MN plus S1, and MN plus S2, respectively. We verified these constructs for SARS-CoV-2 protein expression by western blotting as described above.

### Efficacy study in hamsters

Golden Syrian hamsters (*Mesocricetus auratus*), 9 weeks old, were purchased from Charles River Laboratories. Animals (8/group, equal sex) were immunized ID or IN twice, 3 weeks apart (Week 0 and 3), with 4 × 10^6^ CFU ID or 2 × 10^6^ CFU IN of rLVS Δ*capB*/SCoV2 S, SΔTM, S1, S2, S2E, or MN vaccines diluted in 0.05 ml (ID) or 0.02 ml (IN) sterile phosphate-buffered saline (PBS). The MN vaccine was also administered in combination with the SΔTM or S1 vaccine ID (4 × 10^6^ CFU each) or IN (1 × 10^6^ CFU each). Hamsters vaccinated with PBS (sham) or LVS Δ*capB* (vector) served as controls. Blood was collected 1–3 days prior to each immunization and challenge to assess antibody responses; the sera were heat-inactivated at 56 °C for 30 min. All the animals were challenged IN at Week 8 with ~10^5^ pfu of SARS-CoV-2 (2019-nCoV/USA-WA1/2020 strain) under light anesthesia with ketamine-xylazine. Virus diluted in PBS was administered via pipette into the nares (100 µl total, ~50 µl/nare); animals were observed until fully recovered from anesthesia. Virus back-titration was performed on Vero E6 cells immediately following inoculation, confirming that hamsters received 1.5 (males) or 1.4 (females) × 10^5^ pfu. Hamsters were moved to ABSL3 6–8 days prior to challenge. Animals (*n* = 8/group) were monitored daily post challenge for clinical signs of infection (fever, weight loss, nasal discharge, etc.); weighed on Days 1, 2, 3, 5, and 7 post challenge; and the oropharynx swabbed for virus titers on Days 1, 2, and 3 post challenge. Half the animals (4 hamsters) in each group were euthanized at Day 3 post challenge (acute phase) to assess lung (cranial and caudal lobes) virus titer, which peaks at Day 3, and the other half of each group euthanized at Day 7 (subacute phase) post challenge to evaluate histopathology, which peaks at that time.

### Histopathology assessment

Tissues from hamsters were fixed in 10% buffered formalin for 7–14 days, embedded in paraffin, and cut sections stained with hematoxylin and eosin. Slides were read by a single veterinary pathologist blinded to the identity of the vaccine groups. Cranial and caudal lung histopathology were separately scored for: overall lesion extent, bronchitis, alveolitis, pneumocyte hyperplasia, vasculitis, and interstitial inflammation, each on a 0–4 or 0–5 scale as described in Supplementary Table 2, and the scores for each lung summed.

### Percent alveolar air space quantitation

Hematoxylin and Eosin stained lung sections, 2–4 sections per animal, of infected tissues were scanned at ×20 magnification using an Olympus VS120 microscope, Hamamatsu ORCA-R2 camera, and Olympus VS-ASW 2.9 software through the Experimental Pathology Facility at Colorado State University. Visiopharm software was used for image analysis to detect and quantify alveolar air space. For each tissue section, a region of interest (ROI) identification algorithm was generated at a low magnification with custom decision forest training and classification to differentiate tissue versus background based on color and area. Alveolar air space was identified within tissue ROIs at a high magnification with an additional custom-made K-means clustering algorithm based on staining intensity, area, and morphological features. This algorithm simultaneously normalized varying tissue areas between tissue sections by identifying and excluding any major airways or vessels from analysis. Percent alveolar air space calculations were integrated into a final data output algorithm and were calculated as a proportion of alveolar air space area detected to total tissue area on each section, excluding the variable morphological features including vessels and major airways. Alveolar air space identification and quantification were then reviewed and edited by a pathologist. Median percent air space was determined for each animal.

### Virus assay

Virus titration was performed on oropharyngeal swabs obtained at 1, 2, and 3 days post challenge and on tissue samples of cranial and caudal lungs obtained at 3 days post challenge by double-overlay plaque assay on Vero E6 cells as previously described^46^. Briefly, fluids or tissue homogenates were serially diluted in Tris-buffered Minimum Essential Medium (MEM) with 1% BSA and inoculated onto confluent monolayers of Vero E6 cells seeded in 6-well cell culture plates; incubated at 37 °C for 45 min; and each well overlaid with 2 ml of MEM containing 2% fetal bovine serum and 0.5% agarose. After 24–30 h incubation at 37 °C in 5% CO_2_, a second overlay identical to the first but containing neutral red dye was added and the infected cells continued to culture. At 48–72 h post-infection, plaques were counted with the aid of a lightbox.

Plaque reduction neutralization assay (PRNT) was performed on heat-inactivated hamster sera as described previously^46^.

### Enzyme-linked immunosorbent assay (ELISA) for anti-SARS-CoV-2 RBD and N antibody in hamster sera

Hamster sera were assayed for IgG and subtype antibodies specific to SARS-CoV-2 S RBD and N protein antigens by ELISA as described by us previously^9^. Briefly, RBD or N proteins (1 µg/ml) were diluted in carbonate/bicarbonate buffer (50 mM NaHCO_3_, 50 mM Na_2_CO_3_) and 0.1 ml was used to coat 96-well high-binding capacity plates (Corning, NY) overnight at 4 °C. Excess antigen was removed and residual antigen blocked in Blocker Casein in PBS [Thermo Scientific] for 1 h at room temperature. Sera at a starting dilution of 1:60 were diluted further through a three-fold series with PBS containing 1% bovine serum albumin. The diluted sera were incubated with antigens coated on 96-well plates for 90 min, then incubated for 90 min with horseradish peroxidase (HRP)-conjugated mouse anti-hamster IgG (ThermoFisher), IgG1, or IgG2 (Southern Biotech) at a dilution of 1:1000 at ambient temperature. The plates were washed three times with PBS containing 0.05% Tween-20 after each incubation. One hundred microliters of TMB (3, 3′, 5, 5′ tetramethylbenzidine) substrate in peroxide solution was added to each well and incubated for 15–20 min. The reaction was stopped by adding 100 µl of 2 M sulfuric acid and the solutions were read at 450 nm for absorbance, using a multiscan microplate reader (TiterTek, Huntsville, AL). The endpoint antibody titer is defined as the log_10_ value of the reciprocal of the highest serum dilution that yields an OD greater than the mean OD of sham-immunized control sera plus three standard deviations at the same serum dilution. The results are presented as the mean antibody endpoint titer and SE of the mean (SEM).

### Immunogenicity study in mice

Six- to eight-week-old specific-pathogen-free female BALB/c mice were purchased from The Jackson Laboratory (Sacramento, CA). The mice were immunized ID twice, 3 weeks apart, with normal saline (sham control), 2 × 10^6^ CFU LVS Δ*capB* (vector control), or 2 × 10^6^ CFU of rLVS Δ*capB*/SΔTM, S1, S2 or MN or with rLVS Δ*capB*::MN/SΔTM, S1 or S2. One week after the second immunization, mice were anesthetized by intraperitoneal injection of Ketamine (10 mg/ml)−Xylazine (1 mg/ml) solution, bled, and subsequently euthanized by inhalation of CO_2_. Their spleens and lungs were removed and single-cell suspensions of spleen and lung cells prepared as described by us previously^2,3^. Sera were isolated, heat-inactivated, and frozen at −80 °C until use. T-cell-mediated immune responses were examined by incubating single-cell suspensions of lung and spleen cells with T-cell medium comprising Advanced RPMI-1640 (Invitrogen) supplemented with 2% heat-inactivated (HI) fetal bovine serum (Seradigm Premium Grade), penicillin (100 I.U./ml), streptomycin (100 µg/ml), 0.1 mM non-essential amino acids, 4 mM L-glutamine, 1 mM sodium pyruvate, and 0.05 mM β-mercaptoethanol in the absence and presence of various SARS-CoV-2 and *F. tularensis* antigens; assaying for mouse interferon-gamma (IFN-γ); and quantitating intracellular cytokine staining by flow cytometry analysis. Humoral immune responses were examined by analyzing sera for levels of IgG and subtypes IgG1 and IgG2a antibodies specific for SARS-CoV-2 S RBD, N protein, and HI-LVS^51^.

### Enzyme-linked immunosorbent assay (ELISA) for anti-SARS-CoV-2 antibody in mouse sera

Mouse sera were assayed for IgG and subtype antibodies specific to SARS-CoV-2 S RBD and N protein antigens and to HI-LVS antigens similarly to what is described above for hamster serum with the following modifications. In addition to RBD and N protein antigens, wells were coated with SARS-CoV-2 S (1 µg/ml) or HI-LVS (5 × 10^6^/ml) diluted in carbonate/bicarbonate buffer. Sera at a starting dilution of 1:20 were diluted further through a three-fold series with PBS. After diluted sera were incubated with antigens coated on 96-well plates, the wells were incubated for 90 min with alkaline phosphatase (AP)-conjugated goat anti-mouse IgG (Sigma, St. Louis, MO), IgG1, or IgG2a (Invitrogen) at a dilution of 1:1000 at ambient temperature. After the plates were washed, 100 μl of NPP (*p*-nitrophenylphosphate) substrate in diethanolamine buffer (Phosphatase Substrate kit, BioRad, Hercules, CA) was added to each well and incubated for 15–20 min. The reaction was stopped by adding 100 µl of 0.1 N sodium hydroxide and the solutions were read at 415 nm for absorbance.

### In vitro stimulation and production of IFN-γ by murine immune splenocytes

A single-cell suspension of 1.0 × 10^5^ splenocytes per well was seeded in U-bottom 96-well plates and incubated with T-cell medium alone, or T-cell medium supplemented with 2 µg/mL of recombinant SARS-CoV-2 protein or peptide antigens for 3 days. Afterward, the culture supernatant fluid was collected, cell debris removed by centrifugation, and the supernatant fluid stored in assay diluent (BD Biosciences) at −80 °C until use. The production of mouse IFN-γ in the culture supernatant fluid was assayed using a mouse cytokine EIA kit (BD Biosciences).

### Statistics

In the hamster challenge experiment, body weight means on Days 3–7 post challenge and log_10_ PFU in the oropharyngeal swabs on Days 1–3 post challenge were compared among groups over time via repeated measure analysis of variance (ANOVA) models (mixed models). Mean log_10_ PFU in the cranial or caudal lungs on Day 3 post challenge; mean histopathological scores in cranial and caudal lungs; and mean percent alveolar air space and mean percent alveolar air space change compared with Sham on Day 7 post challenge were compared among groups using one-way ANOVA model. Normal quantile plots of the residual errors (not shown) confirm that the errors follow a normal distribution, as is required when using a parametric model. Analyses were carried out using R version 3.5.2 (R project for statistical computing) and JMP Pro version 15 (SAS Inc., Cary, NC). A linear regression was used to compute the correlation (*r*) between mean N protein-specific serum IgG antibody endpoint titer pre-challenge and mean lung (cranial and caudal) histopathological score at Day 7 post challenge and also between mean histopathological score and mean percent alveolar air space at Day 7 post challenge. Mean and standard error of the mean of serum antibody endpoint titer are reported, and means compared across groups by two-way analysis of variance (ANOVA) with Tukey’s correction for multiple comparisons test using GraphPad Prism, 8.4.3 (San Diego, CA). In the mouse immunology experiments, the sample sizes for assaying immune responses post-vaccination in mice (4/group) were estimated based on previous studies with 80% power using the alpha = 0.05 (i.e., *p* < 0.05) significance criterion. Mean and standard error of serum antibody endpoint titer and cytokine production are shown. Means are compared across groups by one-way or two-way analysis of variance (ANOVA) with Tukey’s correction for multiple comparisons test using GraphPad Prism, 8.4.3 (San Diego, CA).

### Reporting summary

Further information on research design is available in the Nature Research Reporting Summary linked to this article.

## Supplementary information

Supplementary Information

Reporting Summary

## Data Availability

All data supporting the findings of this study are available within the article and its supplementary information files or from the corresponding author upon request.

## References

[1] Center, J. H. C. R. https://coronavirus.jhu.edu/map.html (2020).

[2] Jia Q (2016). *Francisella tularensis* Live Vaccine Strain deficient in *capB* and overexpressing the fusion protein of IglA, IglB, and IglC from the *bfr* promoter induces improved protection against *F. tularensis* respiratory challenge. Vaccine.

[3] Jia Q (2018). Single vector platform vaccine protects against lethal respiratory challenge with Tier 1 select agents of anthrax, plague, and tularemia. Sci. Rep..

[4] Conlan JW (2004). Vaccines against *Francisella tularensis*—past, present and future. Expert Rev. Vaccine.

[5] Mulligan MJ (2017). Tularemia vaccine: Safety, reactogenicity, “Take” skin reactions, and antibody responses following vaccination with a new lot of the *Francisella tularensis* live vaccine strain—A phase 2 randomized clinical Trial. Vaccine.

[6] Clemens DL, Ge P, Lee BY, Horwitz MA, Zhou ZH (2015). Atomic structure of T6SS reveals interlaced array essential to function. Cell.

[7] Clemens DL, Lee BY, Horwitz MA (2004). Virulent and avirulent strains of *Francisella tularensis* prevent acidification and maturation of their phagosomes and escape into the cytoplasm in human macrophages. Infect. Immun..

[8] Clemens DL, Lee BY, Horwitz MA (2005). *Francisella tularensis* enters macrophages via a novel process involving pseudopod loops. Infect. Immun..

[9] Jia Q (2010). A *Francisella tularensis* live vaccine strain (LVS) mutant with a deletion in capB, encoding a putative capsular biosynthesis protein, is significantly more attenuated than LVS yet induces potent protective immunity in mice against F. tularensis challenge. Infect. Immun..

[10] Zhou P (2020). A pneumonia outbreak associated with a new coronavirus of probable bat origin. Nature.

[11] Wan, Y., Shang, J., Graham, R., Baric, R. S. & Li, F. Receptor recognition by the novel coronavirus from wuhan: an analysis based on decade-long structural studies of SARS coronavirus. *J. Virol.* **94**10.1128/JVI.00127-20 (2020). 10.1128/JVI.00127-20 PMC7081895 31996437

[12] Chan JF (2020). Simulation of the clinical and pathological manifestations of Coronavirus Disease 2019 (COVID-19) in a golden Syrian hamster model: implications for disease pathogenesis and transmissibility. Clin. Infect. Dis..

[13] Walls AC (2020). Structure, function, and antigenicity of the SARS-CoV-2 spike glycoprotein. Cell.

[14] Chumakov K, Benn CS, Aaby P, Kottilil S, Gallo R (2020). Can existing live vaccines prevent COVID-19?. Science.

[15] Curtis N, Sparrow A, Ghebreyesus TA, Netea MG (2020). Considering BCG vaccination to reduce the impact of COVID-19. Lancet.

[16] Escobar LE, Molina-Cruz A, Barillas-Mury C (2020). BCG vaccine protection from severe coronavirus disease 2019 (COVID-19). Proc. Natl Acad. Sci. USA.

[17] Hensel J (2020). Protection against SARS-CoV-2 by BCG vaccination is not supported by epidemiological analyses. Sci. Rep..

[18] Imai M (2020). Syrian hamsters as a small animal model for SARS-CoV-2 infection and countermeasure development. Proc. Natl Acad. Sci. USA.

[19] Grifoni A (2020). A sequence homology and bioinformatic approach can predict candidate targets for immune responses to SARS-CoV-2. Cell Host Microbe.

[20] Grifoni A (2020). Targets of T cell responses to SARS-CoV-2 coronavirus in humans with COVID-19 disease and unexposed individuals. Cell.

[21] van Doremalen N (2020). ChAdOx1 nCoV-19 vaccine prevents SARS-CoV-2 pneumonia in rhesus macaques. Nature.

[22] Yu J (2020). DNA vaccine protection against SARS-CoV-2 in rhesus macaques. Science.

[23] Gao Q (2020). Development of an inactivated vaccine candidate for SARS-CoV-2. Science.

[24] Mercado NB (2020). Single-shot Ad26 vaccine protects against SARS-CoV-2 in rhesus macaques. Nature.

[25] Corbett KS (2020). Evaluation of the mRNA-1273 vaccine against SARS-CoV-2 in nonhuman primates. N. Engl. J. Med..

[26] Feng L (2020). An adenovirus-vectored COVID-19 vaccine confers protection from SARS-COV-2 challenge in rhesus macaques. Nat. Commun..

[27] Brocato RL (2021). Protective efficacy of a SARS-CoV-2 DNA vaccine in wild-type and immunosuppressed Syrian hamsters. NPJ Vac..

[28] Dormitzer, P. R., Mandl, C. W. & Rappuoli, R. *Replicating Vaccines* (Springer Basel AG, 2011).

[29] KuoLee R, Harris G, Conlan JW, Chen W (2007). Oral immunization of mice with the live vaccine strain (LVS) of *Francisella tularensis* protects mice against respiratory challenge with virulent type A *F. tularensis*. Vaccine.

[30] Hornick RB, Dawkins AT, Eigelsbach HT, Tulis JJ (1966). Oral tularemia vaccine in man. Antimicrob. Agents Chemother..

[31] Tulis JJ, Eigelsbach HT, Hornick RB (1969). Oral vaccination against tularemia in the monkeys. Proc. Soc. Exp. Biol. Med..

[32] Ray HJ (2009). Oral live vaccine strain-induced protective immunity against pulmonary *Francisella tularensis* challenge is mediated by CD4(+) T cells and antibodies, including immunoglobulin A. Clin. Vaccine Immunol..

[33] Saslaw S, Eigelsbach HT, Prior JA, Wilson HE, Carhart S (1961). Tularemia vaccine study. II. Respiratory challenge. Arch. Intern. Med..

[34] Hornick RB, Eigelsbach HT (1966). Aerogenic immunization of man with live Tularemia vaccine. Bacteriol. Rev..

[35] El Sahly HM (2009). Safety, reactogenicity and immunogenicity of *Francisella tularensis* live vaccine strain in humans. Vaccine.

[36] Eigelsbach HT., H. R. in *Occupational tularemia* Vol. 124, 285–302 (Univ. of Michigan Continued Education, 1962).

[37] Command, U. S. A. M. R. a. D. *Safety and Immunogenicity Study of a Live Francisella Tularensis Vaccine*, https://clinicaltrials.gov/ct2/show/NCT00584844 (2008).

[38] Command, U. S. A. M. R. a. D. *Continued Safety and Immunogenicity Study of a Live Francisella Tularensis Vaccine*, https://clinicaltrials.gov/ct2/show/NCT00787826 (2008).

[39] (NIAID), N. I. o. A. a. I. D. *Phase II Tularemia Vaccine Comparison*, https://clinicaltrials.gov/ct2/show/NCT01150695 (2010).

[40] Salomonsson E (2009). Reintroduction of two deleted virulence loci restores full virulence to the live vaccine strain of *Francisella tularensis*. Infect. Immun..

[41] Wayne Conlan J, Oyston PC (2007). Vaccines against *Francisella tularensis*. Ann. N. Y. Acad. Sci..

[42] Roper RL, Rehm KE (2009). SARS vaccines: where are we?. Expert Rev. Vaccines.

[43] See RH (2008). Severe acute respiratory syndrome vaccine efficacy in ferrets: whole killed virus and adenovirus-vectored vaccines. J. Gen. Virol..

[44] Zhao J, Zhao J, Van Rooijen N, Perlman S (2009). Evasion by stealth: inefficient immune activation underlies poor T cell response and severe disease in SARS-CoV-infected mice. PLoS Pathog..

[45] Lee JS (2006). Mucosal immunization with surface-displayed severe acute respiratory syndrome coronavirus spike protein on Lactobacillus casei induces neutralizing antibodies in mice. J. Virol..

[46] Bosco-Lauth AM (2020). Experimental infection of domestic dogs and cats with SARS-CoV-2: Pathogenesis, transmission, and response to reexposure in cats. Proc. Natl Acad. Sci. USA.

[47] Becker S, Lochau P, Jacob D, Heuner K, Grunow R (2016). Successful re-evaluation of broth medium T for growth of *Francisella tularensis* ssp. and other highly pathogenic bacteria. J. Microbiol. Methods.

[48] Wrapp D (2020). Cryo-EM structure of the 2019-nCoV spike in the prefusion conformation. Science.

[49] Pallesen J (2017). Immunogenicity and structures of a rationally designed prefusion MERS-CoV spike antigen. Proc. Natl Acad. Sci. USA.

[50] LoVullo ED, Molins-Schneekloth CR, Schweizer HP, Pavelka MS (2009). Single-copy chromosomal integration systems for *Francisella tularensis*. Microbiology.

[51] Jia Q, Lee BY, Clemens DL, Bowen RA, Horwitz MA (2009). Recombinant attenuated *Listeria monocytogenes* vaccine expressing *Francisella tularensis* IglC induces protection in mice against aerosolized Type A *F. tularensis*. Vaccine.

