# Supplementary material for: Replicating bacterium-vectored vaccine expressing SARS-CoV-2 Membrane and Nucleocapsid proteins protects against severe COVID-19-like disease in hamsters
Source: NPJ Vaccines. 2021 Mar 30;6:47. doi: 10.1038/s41541-021-00321-8 (PMC8009914; doi:10.1038/s41541-021-00321-8)
Supplement: Supplementary file 1 — Supplementary Information [file 41541_2021_321_MOESM1_ESM.pdf]

Supplementary Information

Replicating bacterium-vectored vaccine expressing SARS-CoV-2 Membrane and Nucleocapsid proteins protects against severe COVID-19-like disease in hamsters

Qingmei Jia[1], Helle Bielefeldt-Ohmann[2], Rachel Maison[3], Saša Masleša-Galić[1], Sarah K. Cooper[4], Richard Bowen[3] and Marcus A. Horwitz[1,*]

[1] Division of Infectious Diseases, Department of Medicine, 37-121 Center for Health Sciences, School of Medicine, University of California – Los Angeles, 10833 Le Conte Avenue, Los Angeles, CA 90095-1688

[2] School of Veterinary Science, University of Queensland, Gatton Campus, Qld 4343, Brisbane, Australia

[3] Department of Biomedical Sciences, Colorado State University, Fort Collins, CO 80523

[4] Department of Microbiology, Immunology, and Pathology, Colorado State University, Fort Collins, CO 80523

Supplementary Figure 1

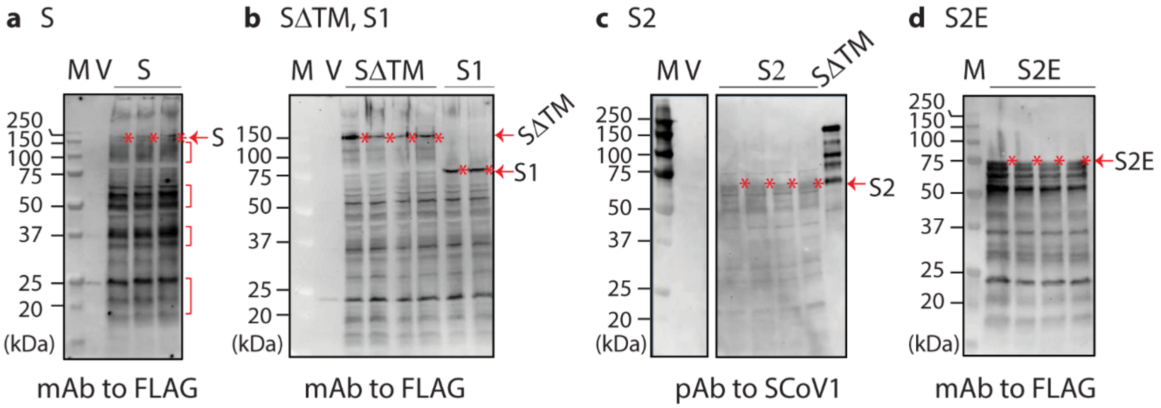

Supplementary Figure 1. Expression of SARS-CoV-2 S, SΔTM, S1, S2, and S2E proteins by rLVS Δ*capB*/SCoV2 vaccines. Expression of SARS-CoV-2 S (**a,** right 3 lanes), SΔTM (**b**, middle 4 lanes), S1 (**b**, right two lanes), S2 (**c**, right panel, left 4 lanes), and S2E (**d**, right 4 lanes) proteins by rLVS Δ*capB*/SCoV2 vaccines. Total bacterial lysates of LVS Δ*capB* vector (V, panels **a**, **b**, and **c**) and rLVS Δ*capB*/SCoV2 vaccines (**a**, **b**, **c** right panel, and **d**) were analyzed by SDS-PAGE and Western blotting with an anti-FLAG monoclonal antibody (**a, b, d**) or an anti-SARS-CoV-1 guinea pig polyclonal antibody (BEI Resources, NR-10361) (**c**). The proteins of interest are indicated by red asterisks to the right of the protein band. The SARS-CoV-1 protein SΔTM (BEI Resources, NR-722, ~150 kDa) (**c**, the rightmost lane) served as a positive control and was also detected by guinea pig polyclonal antibody against SARS-CoV-1. The left and right panels in **c** are from the same gel (Supplementary Fig. 7). All blots are from the same experiment and processed in parallel. All panels: M, protein standards (lane 1); V, LVS Δ*capB* vector; the sizes of the molecular weight markers are labeled to the left of the panels.

Supplementary Figure 2

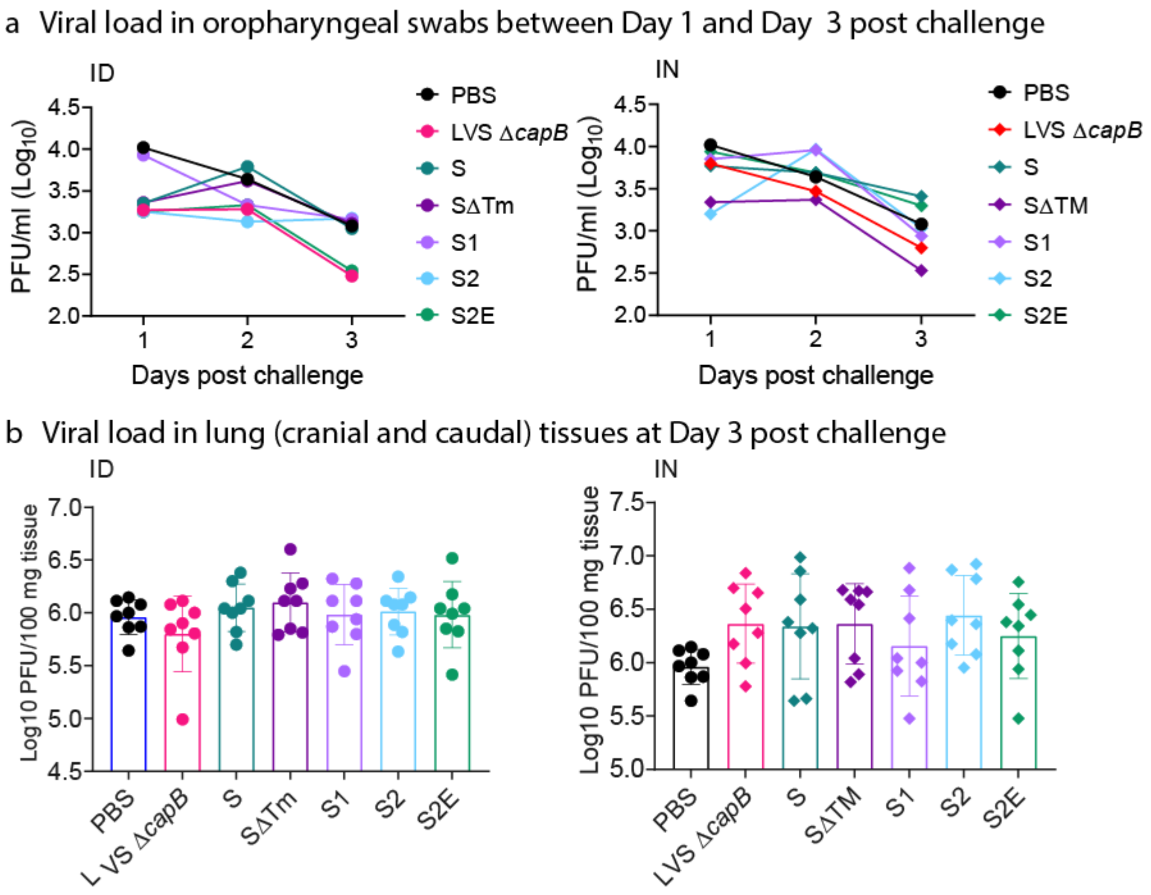

Supplementary Figure 2. Viral load in oropharyngeal swabs and cranial and caudal lungs of hamsters immunized with non-MN vaccines. Hamsters were immunized ID or IN as described in **Fig. 2**. **a.** Oropharyngeal swabs were collected at 1, 2, and 3 days post challenge and assayed for viral load by plaque assay. Data are Mean Log_{10} PFU per ml. The standard errors were omitted in the graphs for clarity. **b.** Cranial and caudal lung homogenates were prepared at Day 3 post challenge and assayed for viral titer. Each symbol represents one animal. Data are of Log_{10} PFU per 100 mg of homogenized tissue (mean ± SE) for each vaccine, as indicated.

Supplementary Figure 3

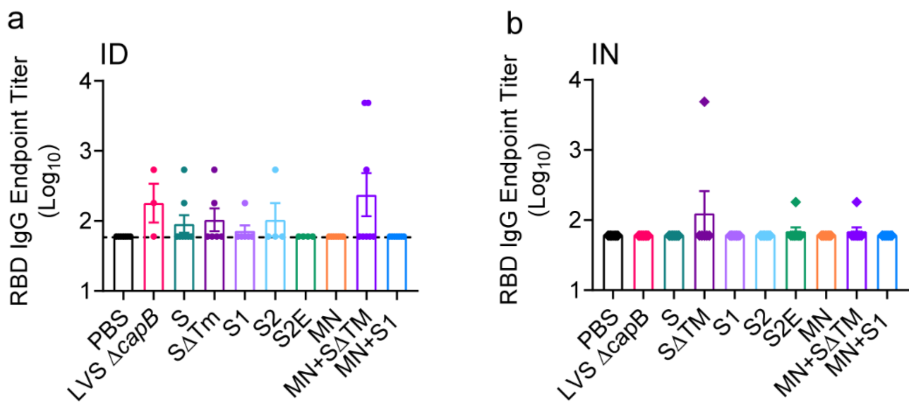

Supplementary Figure 3. RBD-specific humoral immune response induced by non-MN vaccines at Week 8. Hamsters (n = 8 per group, equal sex) were immunized ID (a) or IN (b) twice, 3 weeks apart. At Week 8, the animals were bled and serum evaluated for IgG specific to RBD. Each symbol represents one animal. Data are mean $\log_{10}$ endpoint titer ± SE**.**

Supplementary Figure 4

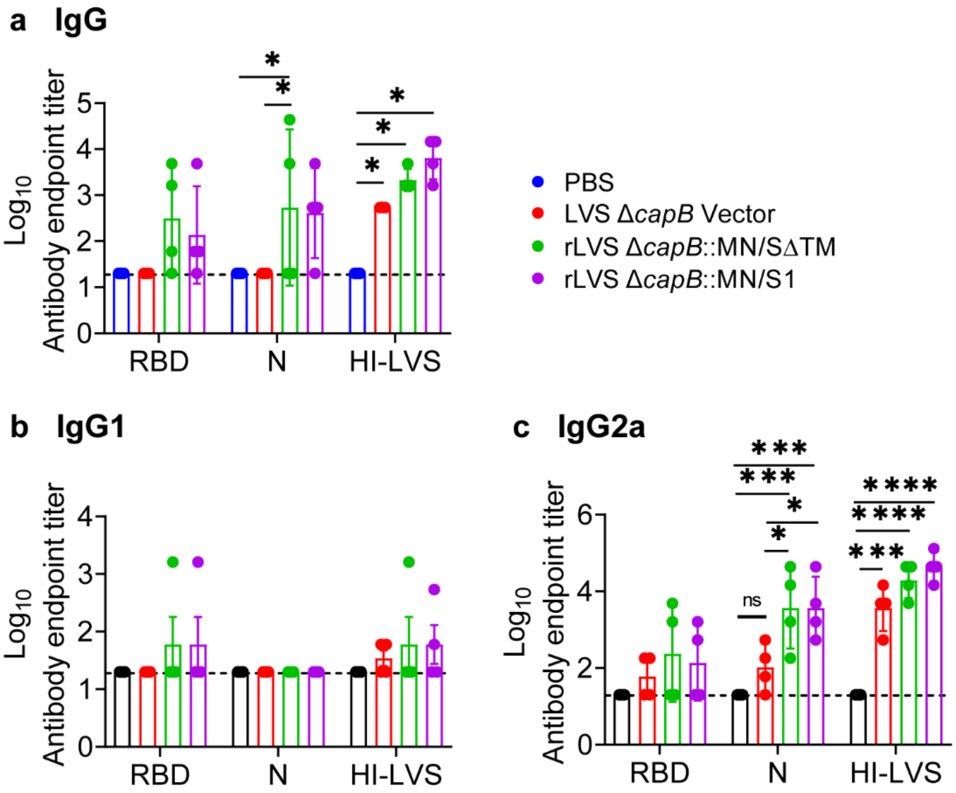

Supplementary Figure 4. Humoral immune response induced by 2nd Generation rLVS Δ*capB*-vectored SARS-CoV2 vaccines in mice. BALB/c mice (n=4/group) were sham-immunized or immunized ID twice at Week 0 and 3 with LVS Δ*capB* vector or 2nd generation rLVS Δ*capB*::MN/SCoV2/SΔTM and rLVS Δ*capB*::MN/SCoV2/S1 expressing the MN protein from the chromosome and the SΔTM and S1 proteins from a shuttle plasmid, as indicated to the right of the graph in **a**. At Week 4, mice were bled and sera assayed for IgG (**a**), IgG1 (**b**) and IgG2a (**c**) specific to SARS-CoV-2 S RBD or N protein or to heat-inactivated LVS Δ*capB* (HI-LVS). Each symbol represents one animal. Data are mean $\log_{10}$ endpoint titer ± SE**.** *, P < 0.05, ***, P<0.001, ****P<0.0001 by Two-way ANOVA with Tukey's multiple comparisons (GraphPad Prism 8.02).

Supplementary Figure 5

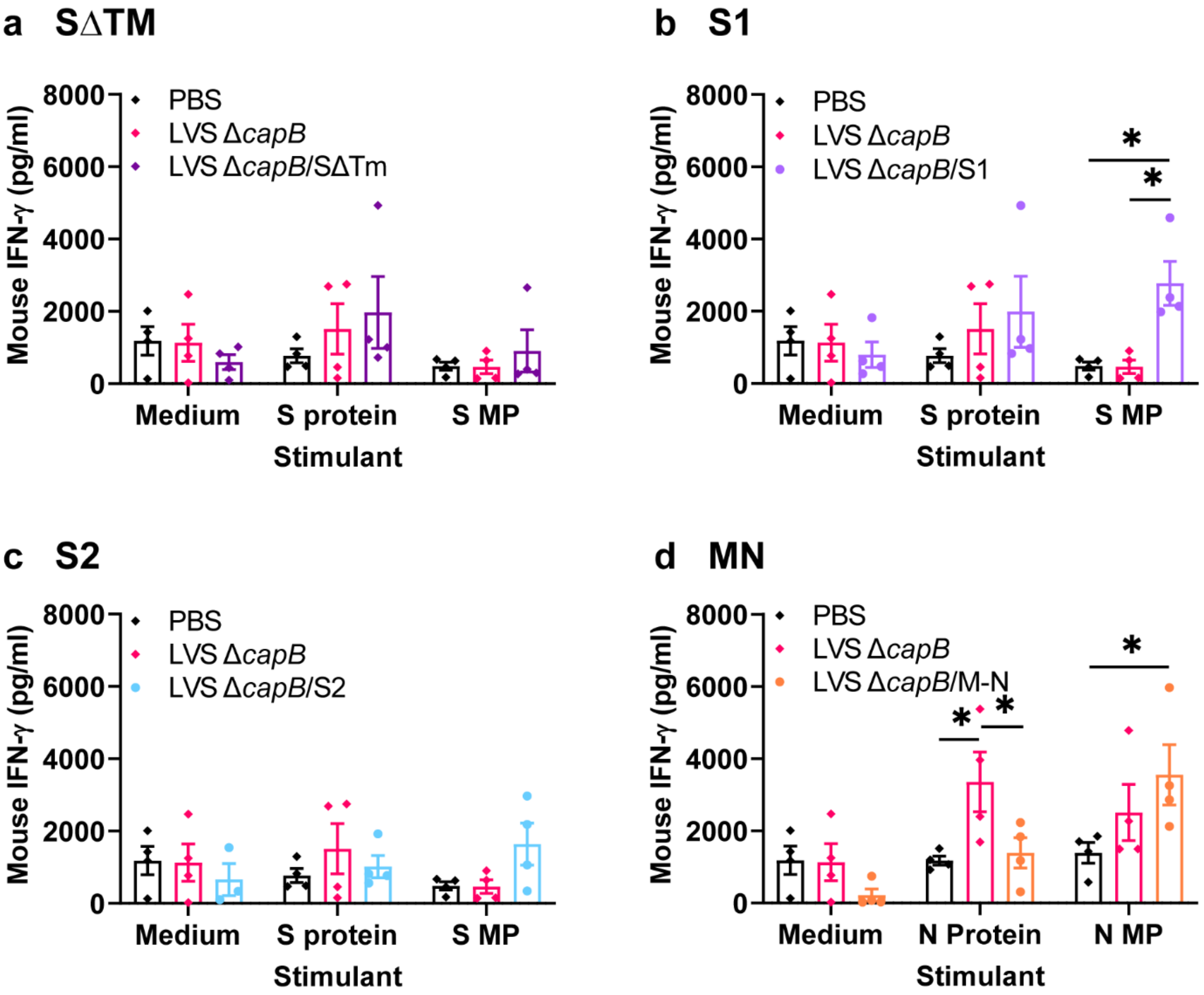

Supplementary Figure 5. Cell-mediated immune responses induced by rLVS Δ*capB*-vectored SARS-CoV-2 vaccines in mice. BALB/c mice (n=4/group) were immunized ID at Week 0 and 3 with SΔTM (**a**), S1 (**b**), S2 (**c**) or MN (**d**); euthanized at Week 4; their splenocytes prepared, stimulated without protein (Medium) or with S or N proteins or the S and N protein master peptide pools (MP) in duplicate or triplicate for 3 days; and culture supernatants collected and assayed for IFN-γ secretion. Each symbol represents one animal. Data are mean IFN-γ secretion ± SE. *, $P < 0.05$ by Two-way ANOVA with Tukey's multiple comparisons (GraphPad Prism 9.0.0).

Supplementary Figure 6

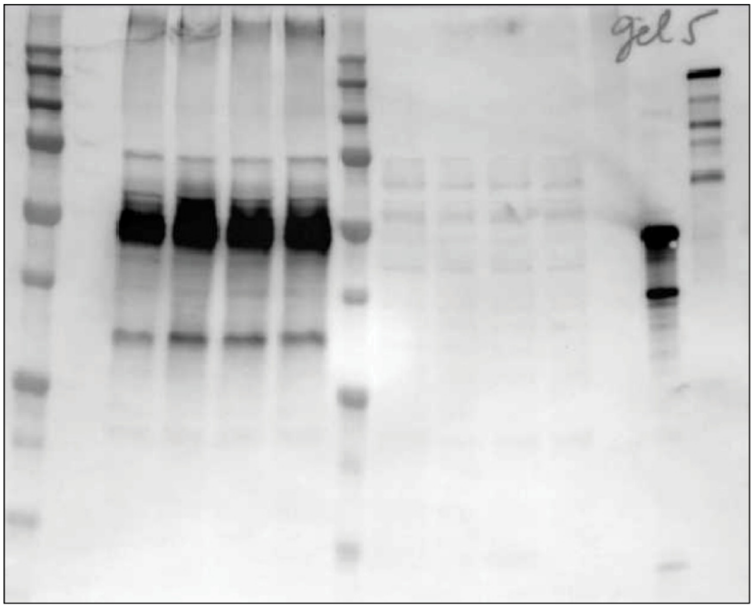

Supplementary Figure 6. Full image of blot shown in Figure 1c**.**

Supplementary Figure 7

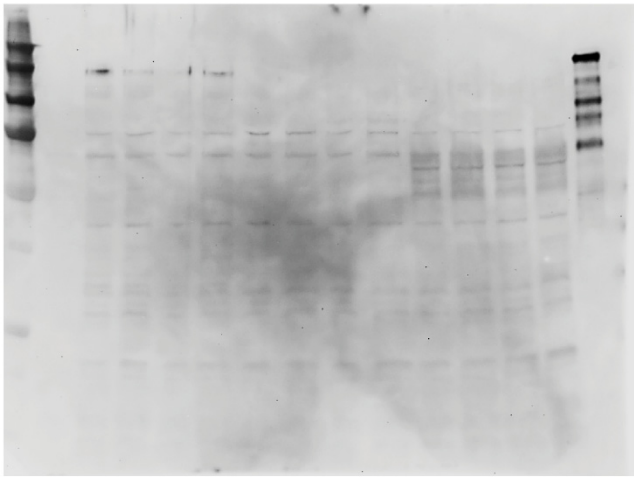

Supplementary Figure 7. Full image of blot shown in Supplementary Figure 1c**.**

Supplementary Table 1. Key statistical analyses of impact of vaccines on weight loss and histopathology

a Weight Loss

Comparison Groups	ID	IN	ID/IN
**Sham vs. MN**	<0.0001	<0.01	<0.0001
**Sham vs. MN+SΔTM**	<0.004	0.001	0.0003
**Sham vs. MN+S1**	0.01	0.0002	0.0002
**Sham vs. All MN Groups**	0.0001	0.0001	<0.0001
**All MN vs. All S protein Groups**	0.0008	0.0001	<0.0001

b Histopathology - Combined Cranial and Caudal Lung Scores

Comparison Groups	ID	IN	ID/IN
**Sham vs. MN**	<0.0001	<0.0001	<0.0001
**Sham vs. MN+SΔTM**	<0.0001	<0.0001	<0.0001
**Sham vs. MN+S1**	<0.0001	<0.0001	<0.0001
**Sham vs. All MN Groups**	<0.001	<0.001	<0.0001
**All MN vs. All S protein Groups**	<0.001	<0.001	<0.0001

Means of each vaccine group Day 7 post-challenge were compared using a repeated measure (mixed) analysis of variance model. Normal quantile plots of the residual errors (not shown) confirm that the errors follow a normal distribution, as is required when using a parametric repeated measure model. ID, intradermal route of vaccination; IN, intranasal route of vaccination; ID/IN, intradermal or intranasal route of vaccination. All MN Groups, groups vaccinated with the rLVS Δ*capB*/SCoV2 vaccine expressing MN alone or combined with an rLVS Δ*capB*/SCoV2 vaccine expressing SΔTM or S1. All S Protein Groups, groups vaccinated with rLVS Δ*capB*/SCoV2 vaccine expressing S, SΔTM, S1, S2, or S2-E.

Supplementary Table 2. Histopathological Score criteria for SARS-CoV-2 infection in hamsters

Score	Overall Lesion Extent	Bronchitis Bronchiolitis	Alveolitis (alveolar inflammation: includes transudates, exudates and/or hemorrhage)	Pneumocyte hyperplasia	Vasculitis***	Interstitial Inflammation including perivascular
**0**	No lesion apparent	no change	no change	none apparent	no change	no change
**1**	< 10%	minimal cellular debris in lumen	minimal change*	minimal change**	minimal change*	minimal change*
**2**	11 – 30%	mild cellular debris or effete macrophages in lumen	mild change	mild change	mild change	mild change
**3**	31 – 60%	moderate change including transepithelial inflammation	moderate change	moderate change	moderate change	moderate change
**4**	> 60%	severe change in <50% of bronchi/bronchioles with peri- and transmural inflammation	severe change in <50% of lung lobe	severe change in <50% of lung lobe	severe change in <50% of lung lobe	severe change in <50% of lung lobe or other tissue/organ
**5**		severe change in >50% of bronchi/bronchioles with peri- and transmural inflammation	severe change in >50% of lung lobe	severe change in >50% of lung lobe	severe change in >50% of lung lobe	severe change in >50% of lung lobe or other tissue/organ

* or possibly non-specific background or appearance compounded by lung collapse, i.e., not inflated; focal inflammation of limited extent

** or possibly non-specific background or appearance compounded by lung collapse, i.e., not inflated

*** Note: perivascular infiltrates in lung are regarded as interstitial inflammation if no evidence of endothelial involvement is apparent. True vasculitis will involve endothelium, subendothelial layer and media (latter only in case of larger vessels)

Supplementary Table 3. Lung Histopathology Scores at Day 7 post challenge

Hamster	Sex	Overall Lesion Extent	Bronchitis	Alveolitis	Pneumocyte Hyperplasia	Vasculitis	Interstitial Inflammation	Total score
**Cranial**								
A3	M	3	2	4	4	2	4	19
A4	M	4	2	5	5	2	5	23
A7	F	3	1	4	4	2	4	18
A8	F	3	1	4	4	2	4	18
B3	M	3	1	4	3	1	4	16
B4	M	3	1	4	3	3	4	18
B7	F	3	2	4	4	3	4	20
B8	F	3	2	4	4	2	4	19
C3	M	4	2	5	4	2	5	22
C4	M	3	1	4	4	2	4	18
C7	F	3	2	4	4	2	4	19
C8	F	3	2	4	4	2	4	19
D3	M	4	2	5	4	2	5	22
D4	M	3	0	3	2	2	3	13
D7	F	3	2	4	4	2	4	19
D8	F	3	1	4	4	1	4	17
E3	M	3	2	4	4	2	4	19
E4	M	4	3	5	4	2	5	23
E7	F	3	2	4	4	1	4	18
E8	F	3	1	4	4	1	4	17
F7	F	3	2	4	4	1	4	18
F8	F	3	2	4	4	1	4	18
G3	M	3	2	4	4	2	4	19
G4	M	3	2	4	3	3	4	19
G7	F	3	2	4	4	3	4	20
G8	F	3	2	4	4	2	4	19
H3	M	1	0	1	0	0	2	4
H4	M	1	0	0	1	0	2	4
H7	F	1	0	1	1	0	1	4
H8	F	1	0	1	0	2	2	6
I3	M	1	2	0	0	2	2	7
I4	M	1	0	0	0	0	2	3
I5	F	1	0	1	0	3	2	7
I7	F	2	1	1	0	4	2	10
J4	M	2	0	2	2	2	2	10
J7	F	2	2	0	0	3	2	9
J8	F	2	2	2	0	3	2	11
K3	M	3	1	4	4	2	4	18
K4	M	3	1	4	4	1	4	17
K7	F	3	2	4	4	2	4	19
K8	F	3	1	4	4	1	4	17
L3	M	3	1	4	4	2	4	18
L4	M	3	2	4	4	1	4	18
L7	F	3	2	4	4	2	4	19
L8	F	3	2	4	4	2	4	19

Supplementary Table 3 Continued

Hamster	Sex	Overall Lesion Extent	Bronchitis	Alveolitis	Pneumocyte Hyperplasia	Vasculitis	Interstitial Inflammation	Total score
M3	M	3	1	3	3	2	3	15
M4	M	3	1	4	4	2	4	18
N3	M	4	2	5	5	1	5	22
N4	M	3	2	4	4	2	4	19
N7	F	3	2	4	4	2	4	19
N8	F	3	2	4	4	3	4	20
O3	M	4	1	5	5	2	5	22
O4	M	2	0	3	3	0	3	11
O8	F	3	1	4	4	2	4	18
P3	M	3	2	4	4	1	4	18
P4	M	3	1	4	4	1	4	17
P7	F	3	2	4	4	3	4	20
P8	F	3	2	4	4	1	4	18
Q3	M	2	0	0	0	0	2	4
Q4	M	2	0	1	0	3	2	8
Q7	F	1	0	1	1	0	1	4
Q8	F	2	2	2	0	2	2	10
R3	M	2	1	2	2	1	2	10
R4	M	2	0	1	1	2	2	8
R7	F	2	2	2	2	2	2	12
R8	F	2	1	2	2	2	2	11
S4	M	1	0	0	0	1	1	3
S7	F	1	1	0	0	3	2	7
S8	F	2	1	3	3	2	3	14
**Caudal**								
A3	M	4	2	4	4	3	4	21
A4	M	3	2	4	4	2	4	19
A7	F	3	1	4	4	1	4	17
A8	F	3	2	4	4	2	4	19
B3	M	3	2	4	4	2	4	19
B4	M	4	1	5	5	1	5	21
B7	F	3	2	4	4	3	4	20
B8	F	3	2	4	4	2	4	19
C3	M	3	2	4	3	2	4	18
C4	M	3	2	4	4	2	4	19
C7	F	3	2	4	4	3	4	20
C8	F	3	2	4	4	2	4	19
D3	M	4	2	4	4	2	4	20
D4	M	3	2	4	4	2	4	19
D7	F	3	2	4	4	2	4	19
D8	F	3	3	4	4	2	4	20
E3	M	3	1	4	4	2	4	18
E4	M	4	3	5	4	2	5	23
E7	F	3	2	4	4	2	4	19
E8	F	3	0	4	4	2	4	17
F7	F	3	1	4	4	2	4	18
F8	F	3	2	4	4	2	4	19

Supplementary Table 3 Continued

Hamster	Sex	Overall Lesion Extent	Bronchitis	Alveolitis	Pneumocyte Hyperplasia	Vasculitis	Interstitial Inflammation	Total score
G3	M	4	3	5	4	3	5	24
G4	M	4	2	5	4	4	5	24
G7	F	2	2	4	4	2	4	18
G8	F	3	2	4	4	4	4	21
H3	M	1	0	0	0	0	2	3
H4	M	1	0	2	2	1	2	8
H7	F	1	0	2	2	0	2	7
H8	F	2	2	1	0	1	2	8
I3	M	1	0	0	0	3	2	6
I4	M	2	0	1	0	0	2	5
I5	F	1	0	0	0	3	2	6
I7	F	1	0	0	0	3	2	6
J4	M	1	1	1	2	1	2	8
J7	F	2	0	0	0	2	2	6
J8	F	2	1	2	1	3	2	11
K3	M	3	1	4	3	1	4	16
K4	M	3	1	4	4	1	4	17
K7	F	3	2	3	3	1	3	15
K8	F	3	1	4	4	2	4	18
L3	M	3	1	4	4	2	4	18
L4	M	2	1	3	3	2	3	14
L7	F	3	1	4	4	2	4	18
L8	F	3	2	4	4	1	4	18
M3	M	3	1	4	4	1	4	17
M4	M	3	1	4	4	1	4	17
N3	M	3	1	4	4	2	4	18
N4	M	4	2	5	4	3	5	23
N7	F	3	1	4	4	1	4	17
N8	F	3	2	4	4	1	4	18
O3	M	3	2	4	4	3	4	20
O4	M	3	2	4	4	2	4	19
O8	F	3	1	4	4	2	4	18
P3	M	4	1	4	4	2	4	19
P4	M	3	2	4	4	0	4	17
P7	F	3	2	4	4	2	4	19
P8	F	3	1	4	4	2	4	18
Q3	M	2	0	2	2	0	2	8
Q4	M	1	0	1	2	3	2	9
Q7	F	1	0	2	1	0	1	5
Q8	F	2	1	2	2	0	2	9
R3	M	3	2	3	3	1	3	15
R4	M	2	0	2	2	2	2	10
R7	F	2	2	3	3	1	3	14
R8	F	1	0	1	1	0	1	4
S4	M	2	1	0	0	3	3	9
S7	F	1	1	0	0	2	1	5
S8	F	2	0	3	2	1	3	11
