# Supplementary material for: Replicating bacterium-vectored vaccine expressing SARS-CoV-2 Membrane and Nucleocapsid proteins protects against severe COVID-19-like disease in hamsters
Source: NPJ Vaccines. 2021 Mar 30;6:47. doi: 10.1038/s41541-021-00321-8 (PMC8009914; doi:10.1038/s41541-021-00321-8)
Supplement: Supplementary file 2 — Reporting Summary [file 41541_2021_321_MOESM2_ESM.pdf]

nature research

Corresponding author(s): Marcus A. Horwitz

Last updated by author(s): Mar 8, 2021

Reporting Summary

Nature Research wishes to improve the reproducibility of the work that we publish. This form provides structure for consistency and transparency in reporting. For further information on Nature Research policies, see our Editorial Policies and the Editorial Policy Checklist.

Statistics

For all statistical analyses, confirm that the following items are present in the figure legend, table legend, main text, or Methods section.

n/a	Confirmed	
☐	☒	The exact sample size (*n*) for each experimental group/condition, given as a discrete number and unit of measurement
☐	☒	A statement on whether measurements were taken from distinct samples or whether the same sample was measured repeatedly
☐	☒	The statistical test(s) used AND whether they are one- or two-sided<br>*Only common tests should be described solely by name; describe more complex techniques in the Methods section.*
☒	☐	A description of all covariates tested
☐	☒	A description of any assumptions or corrections, such as tests of normality and adjustment for multiple comparisons
☐	☒	A full description of the statistical parameters including central tendency (e.g. means) or other basic estimates (e.g. regression coefficient) AND variation (e.g. standard deviation) or associated estimates of uncertainty (e.g. confidence intervals)
☐	☒	For null hypothesis testing, the test statistic (e.g. *F*, *t*, *r*) with confidence intervals, effect sizes, degrees of freedom and *P* value noted<br>*Give P values as exact values whenever suitable.*
☒	☐	For Bayesian analysis, information on the choice of priors and Markov chain Monte Carlo settings
☒	☐	For hierarchical and complex designs, identification of the appropriate level for tests and full reporting of outcomes
☒	☐	Estimates of effect sizes (e.g. Cohen's *d*, Pearson's *r*), indicating how they were calculated

Our web collection on statistics for biologists contains articles on many of the points above.

Software and code

Policy information about availability of computer code

Data collection	N/A
Data analysis	R version 3.5.2 (R project for statistical computing) and JMP Pro version 15 (SAS Inc., Cary NC); GraphPad Prism, 8.4.3 (San Diego, CA)

For manuscripts utilizing custom algorithms or software that are central to the research but not yet described in published literature, software must be made available to editors and reviewers. We strongly encourage code deposition in a community repository (e.g. GitHub). See the Nature Research guidelines for submitting code & software for further information.

Data

Policy information about availability of data

All manuscripts must include a data availability statement. This statement should provide the following information, where applicable:
- Accession codes, unique identifiers, or web links for publicly available datasets
- A list of figures that have associated raw data
- A description of any restrictions on data availability

All data supporting the findings of this study are available within the article and its supplementary information files or from the corresponding author upon request.

Field-specific reporting

Please select the one below that is the best fit for your research. If you are not sure, read the appropriate sections before making your selection.

☒ Life sciences ☐ Behavioural & social sciences ☐ Ecological, evolutionary & environmental sciences

For a reference copy of the document with all sections, see nature.com/documents/nr-reporting-summary-flat.pdf

Life sciences study design

All studies must disclose on these points even when the disclosure is negative.

Sample size	The sample sizes for assaying vaccine efficacy post-challenge (8 animals per group) were based on previous studies of LVS dcapB Platform vaccines. The sample sizes for assaying immune responses post-vaccination in mice (4/group) were estimated based on previous studies with 80% power using the alpha = 0.05 (i.e. $p < 0.05$) significance criterion. The sample sizes for assaying immune responses post-vaccination in mice (4/group) were estimated based on previous studies with 80% power using the alpha = 0.05 (i.e. $p < 0.05$) significance criterion.
Data exclusions	No data was excluded.
Replication	Hamster study was conducted once. All vaccines were given both intradermally and intranasally. In addition, the MN vaccine was given both alone and in combination with two other vaccines that by themselves did not work, both intradermally and intranasally. Thus, six groups contained the MN vaccine.
Randomization	Hamsters were assigned to groups randomly.
Blinding	The pathologist was blinded to the vaccine status of each group in the hamster study.

Reporting for specific materials, systems and methods

We require information from authors about some types of materials, experimental systems and methods used in many studies. Here, indicate whether each material, system or method listed is relevant to your study. If you are not sure if a list item applies to your research, read the appropriate section before selecting a response.

Materials & experimental systems

n/a	Involved in the study	
☐	☒	Antibodies
☐	☒	Eukaryotic cell lines
☒	☐	Palaeontology and archaeology
☐	☒	Animals and other organisms
☒	☐	Human research participants
☒	☐	Clinical data
☒	☐	Dual use research of concern

Methods

n/a	Involved in the study	
☒	☐	ChIP-seq
☒	☐	Flow cytometry
☒	☐	MRI-based neuroimaging

Antibodies

Antibodies used	Guinea pig polyclonal anti-SARS coronavirus antibody (NR-10361) was obtained from BEI Resources and monoclonal anti-FLAG M2 HRP antibody was purchased from Millipore Sigma (St. Louis, MO).
Validation	The guinea pig polyclonal anti-SARS coronavirus antibody was validated by Western blotting analysis using SARS-CoV-1 protein SΔTM (BEI Resources, NR-722).

Eukaryotic cell lines

Policy information about cell lines

Cell line source(s)	Vero E6 cells were purchased from ATCC (#CCL-81, Manassas, VA, USA). Used only to assay PFU of virus.
Authentication	The cell line was not authenticated.
Mycoplasma contamination	The cell line was not tested for mycoplasma contamination.
Commonly misidentified lines (See ICLAC register)	N/A

Animals and other organisms

Policy information about studies involving animals; ARRIVE guidelines recommended for reporting animal research

Laboratory animals	Golden Syrian hamsters (Mesocricetus auratus), age 9 weeks, were purchased from Charles River Laboratories); Six to eight week old specific-pathogen-free female BALB/c mice were purchased from Charles River Laboratory (Sacramento, CA).
Wild animals	N/A.
Field-collected samples	N/A.
Ethics oversight	Hamsters and mice were used according to protocols approved by the Institutional Animal Care and Use Committees of Colorado State University (CSU) and UCLA, respectively.

Note that full information on the approval of the study protocol must also be provided in the manuscript.
